# Hematopoietic PBX-interacting protein mediates cartilage degeneration during the pathogenesis of osteoarthritis

**DOI:** 10.1038/s41467-018-08277-5

**Published:** 2019-01-18

**Authors:** Quanbo Ji, Xiaojie Xu, Lei Kang, Yameng Xu, Jingbo Xiao, Stuart B. Goodman, Xiang Zhu, Wenchao Li, Juan Liu, Xu Gao, Zhifeng Yan, Yuxuan Zheng, Zheng Wang, William J. Maloney, Qinong Ye, Yan Wang

**Affiliations:** 1 Department of Orthopaedics, General Hospital of Chinese People’s Liberation Army, 100853 Beijing, China; 2 Department of Medical Molecular Biology, Beijing Institute of Biotechnology, 100850 Beijing, China; 3 Department of Orthopaedic Surgery, Stanford University, Palo Alto, CA 94305 USA; 4 Department of Nuclear Medicine, Peking University First Hospital, 100034 Beijing, China; 5 Department of Traditional Chinese Medicine, Xinhua Hospital Affiliated to Shanghai Jiao Tong University School of Medicine, 200025 Shanghai, China; 6 National Library of China, 100081 Beijing, China; 7 Department of Orthopaedic Surgery, Johns Hopkins University School of Medicine, Baltimore, MD 21205 USA; 8 Peking-Tsinghua Center for Life Sciences, Academy for Advanced Interdisciplinary Studies, School of Life Sciences, Peking University, 100871 Beijing, China

## Abstract

Osteoarthritis (OA) has been recognized as the most common chronic age-related disease. Cartilage degeneration influences OA therapy. Here we report that hematopoietic pre-B cell leukemia transcription factor-interacting protein (*HPIP*) is essential for OA development. Elevated *HPIP* levels are found in OA patients. *Col2a1-CreER*^*T2*^*/HPIP*^f/f^ mice exhibit obvious skeletal abnormalities compared with their *HPIP*^f/f^ littermates. *HPIP* deficiency in mice protects against developing OA. Moreover, intra-articular injection of adeno-associated virus carrying *HPIP*-specific short hairpin RNA in vivo attenuates OA histological signs. Notably, in vitro RNA-sequencing and chromatin immunoprecipitation sequencing profiles identify that *HPIP* modulates OA cartilage degeneration through transcriptional activation of *Wnt* target genes. Mechanistically, *HPIP* promotes the transcription of *Wnt* targets by interacting with lymphoid enhancer binding factor 1 (*LEF1*). Furthermore, *HPIP* potentiates the transcriptional activity of *LEF1* and acetylates histone H3 lysine 56 in the promoters of *Wnt* targets, suggesting that *HPIP* is an attractive target in OA regulatory network.

## Abstract (web summary)

Osteoarthritis (OA) is associated with cartilage degeneration, and no effective therapy exists. Here, the authors show that the HPIP protein modulates OA progression by regulating Wnt signaling, and that its knockdown in joints via AAV-mediated gene silencing attentuates pathology.

## Introduction

Osteoarthritis (OA) is the most common worldwide age-related and post-traumatic degenerative joint disorder^1–3^. According to the statistics of the Centers for Disease Control and Prevention, arthritis affects an estimated 26.0% of women and 19.1% of men who are diagnosed by a doctor in the United States and OA is present in 80% of the population by age 65^4,5^. OA is mainly characterized by a cartilage homeostasis disorder with subsequent inflammation and degradation that results in chronic physical disability and progressive irreversible dysfunction^6–9^. Although accumulating reports have identified factors to predict and modify the development of OA, the clinical efficacy of treatments for cartilage damage and regeneration is still very limited.

Age, metabolism, and mechanical, genetic and environmental factors have gained widespread acceptance as the leading causes of degradation of cartilage extracellular matrix (ECM) molecules, such as collagen and aggrecan (*ACAN*), the loss of function of which is closely related to the progression of OA^10–12^. The maintenance of cartilage integrity and homeostasis depends on its normal structure and components^13–15^. Ultimately, this research will shed light on new therapies for the treatment of cartilage damage and degeneration in OA.

Previously, we and others have shown that hematopoietic pre-B cell leukemia transcription factor-interacting (*PBX*-interacting) protein (*HPIP/PBXIP1*) mainly functions as a modulator of cancer carcinogenesis and progression^16–18^. *HPIP* silencing significantly suppresses migration/invasion and epithelial–mesenchymal transition (EMT) in lung cancer^19^. Stabilization of microtubules represses estrogen receptor α transcriptional activity in a *HPIP*-dependent manner^20^. The *HPIP-ER* complex network in breast cancer promotes cell proliferation and migration in vitro and in vivo^21^. Moreover, *HPIP* has also emerged as a novel substrate of calpain2 and activator of *FAK*^18^, which suggests the significance of *HPIP* in organogenesis and tumorigenesis. Besides, *HPIP* silencing suppressed *TGF-β*-induced EMT by inhibiting *Smad2* activation and *TGF-β* treatment induced *HPIP* expression in cancer cells^22,23^. *TGF-β* is an endogenously pleiotropic cytokine that is known for the important regulation of OA cartilage homeostasis^6^. However, the potential role of *HPIP* in human OA remains unknown.

In this study, using *Col2a1-CreER*^*T2*^*/HPIP*^f/f^ mice as an animal model, we find that *HPIP* is essential for the OA development. *HPIP* deficiency in chondrocyte-specific knockout of *HPIP* in mice protect against OA cartilage degeneration. Besides, intra-articular injection of adeno-associated virus (AAV) carrying *HPIP*-specific short hairpin RNA (shRNA) in vivo attenuates OA development. By combining RNA-sequencing (RNA-seq) and chromatin immunoprecipitation sequencing (ChIP-seq), we identify that *HPIP* modulates OA cartilage degeneration through transcriptional activation of *Wnt* signaling pathway and epigenetic modulation of transcriptional programs, suggesting that these novel functions of HPIP will likely lead to new avenues of OA treatment.

## Results

### Elevated *HPIP* levels in the cartilage of OA patients

**Fig. 1:**
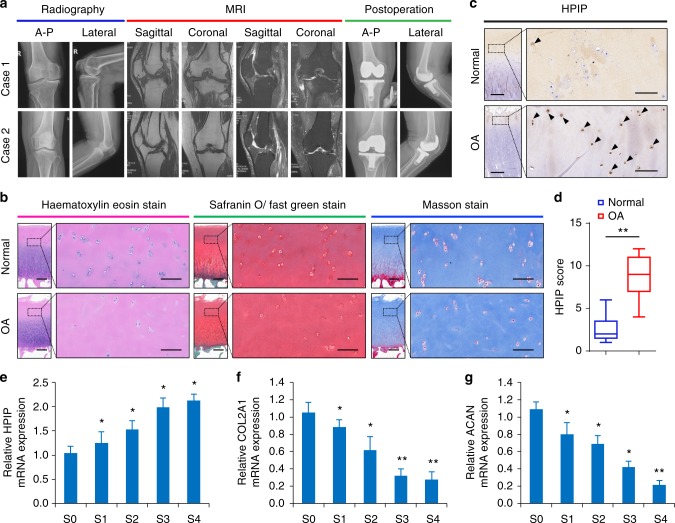
*HPIP* expression is upregulated in osteoarthritis (OA) cartilage. **a** Representative plain radiographs and MRI images of patients with knee OA undergoing knee arthroplasty surgery. A–P anterior–posterior. **b** Representative histopathological staining of normal and OA cartilage tissues. Hematoxylin–eosin, safranin O/fast green and Masson trichrome staining were performed to examine the proteoglycan content in cartilage. Scale bar, left, 500 μm; right, 200 μm. **c** Immunohistochemistry assay with anti-*HPIP* in normal and OA cartilage tissues. Scale bar, left, 500 μm; right, 50 μm. **d** The *HPIP* scores in normal (*n* = 118) and OA (*n* = 118) cartilage tissues based on an immunohistochemistry assay were compared with the Mann–Whitney *U*-test. Center value represents the median of the *HPIP* scores. The bounds of box represent the upper quartile and the lower quartile. The whiskers represent the maximum and minimum score. **e** qRT-PCR measurement of *HPIP* in cartilage tissues obtained from stage 0 (S0) to stage 4 (S4). **f** qRT-PCR measurement of *COL2A1* in cartilage tissues obtained from stage 0 (S0) to stage 4 (S4). **g** qRT-PCR measurement of *ACAN* in cartilage tissues obtained from stage 0 (S0) to stage 4 (S4). Error bar represents the standard deviation (s.d.) and *P-*value was generated by using one-way ANOVA with Tukey’s post hoc test. *P < 0.05; **P < 0.01

To investigate the potential role of *HPIP* in OA, we first examined the expression levels of *HPIP* in 118 pairs of OA cartilage tissues and corresponding non-lesion samples (Fig. 1a). Prior to experimental assessment, we performed hematoxylin–eosin (HE), safranin O/fast green and Masson trichrome staining of the cartilage tissues of each patient (Fig. 1b) and detected the expression of *HPIP* protein using an immunohistochemical staining assay and qRT-PCR in 118 pairs of OA cartilage tissues and corresponding non-lesion samples. *HPIP* expression was significantly higher in OA cartilage than in non-lesion tissues (Fig. 1c–d). To further identify the stage of OA that *HPIP* expression manifests and assess the effects of the *HPIP* on the ECM components, we investigated the expression of *HPIP* and *COL2A1* and *ACAN* at different stages of cartilage tissues using qRT-PCR analysis (Fig. 1e–g). The data revealed that the expression of *HPIP* was gradually enhanced from stage 1 and the sustainable increase in the mRNA levels of the *HPIP* was significantly observed in stage 4 of OA cartilage, whereas the production of *COL2A1* and *ACAN* decreased gradually from stage 1 to the late stage of OA, indicating the potential involvement of *HPIP* in ECM degradation in OA articular cartilage degeneration.

### *HPIP* deficiency impaired articular cartilage development

**Fig. 2:**
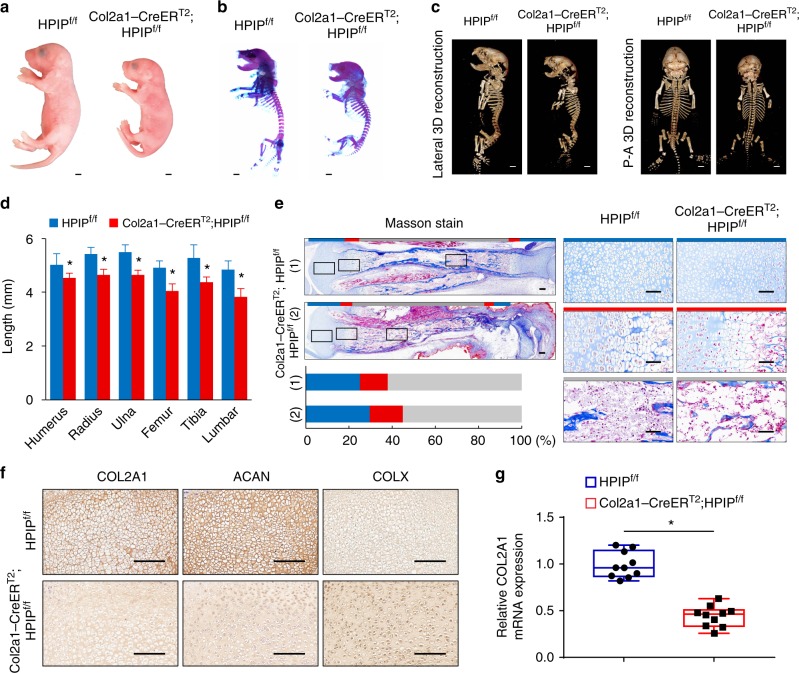
Skeletal and cartilage abnormalities in *Col2a1-CreER*^*T2*^*;HPIP*^f/f^ mice. **a** *Col2a1-CreER*^*T2*^*;HPIP*^f/f^ mice and *HPIP*^f/f^ littermate embryos (P1). Scale bar, 1 mm. **b** Double staining with alizarin red and alcian blue of the whole skeleton. Scale bar, 1 mm. **c** Lateral and posterior–anterior (P–A) views of μCT three-dimensional reconstruction analysis. Scale bar, 1 mm. **d** The length of long bones and vertebra (first to fifth lumbar spines) of *HPIP*^f/f^ (*n* = 10) and *Col2a1-CreER*^*T2*^*;HPIP*^f/f^ (*n* = 10) littermate embryos (P1). **e** Masson trichrome staining of whole tibias. The boxed areas represent the regions of right magnified images with a matched color rim. Scale bar, 100 μm. The percentage of the length of the proliferative zone (blue), hypertrophic zone (red), and bone area (gray) over the total tibia length of *HPIP*^f/f^ (*n* = 10) and *Col2a1-CreER*^*T2*^*;HPIP*^f/f^ (*n* = 10) littermate embryos (P1). **f**, **g** Representative immunohistochemistry assay (**f**) with the indicated antibodies of the proximal tibias of *HPIP*^f/f^ and *Col2a1-CreER*^*T2*^*;HPIP*^f/f^ littermate embryos (P1) and boxplots showing qRT-PCR (**g**) of the proximal tibias of *HPIP*^f/f^ (*n* = 10) and *Col2a1-CreER*^*T2*^*;HPIP*^f/f^ (*n* = 10) littermate embryos (P1). Scale bar, 100 μm. Center value represents the median of the *HPIP* scores. The bounds of box represent the upper quartile and the lower quartile. The whiskers represent the maximum and minimum score. Error bar represents the standard deviation (s.d.) and *P-*value was generated by using one-way ANOVA with Tukey’s post hoc test. *P < 0.05; **P < 0.01

To determine the physiological involvement of endogenous *HPIP* gene in cartilage homeostasis, we next investigated the phenotype of chondrocyte-specific *HPIP* knockout (KO) mice. Pregnant mice with embryos at E12.5 were injected with tamoxifen. The knockdown efficiency of *HPIP* was confirmed by DNA sequencing and immunoblot in *Col2a1-CreER*^*T2*^*/HPIP*^f/f^ mice (Supplementary Fig. 1a, b). Whole skeletal alizarin red, alcian blue staining, μCT and HE staining were performed. The *Col2a1-CreER*^*T2*^*/HPIP*^f/f^ mice showed mild but proportional dwarfism compared to *HPIP*^f/f^ littermates from embryonic stages up to 1 week after birth (Fig. 2a–c and Supplementary Fig. 2a). The lengths of the humerus, radius, ulna, femur, tibia, and vertebrae were 10–21% shorter in *Col2a1-CreER*^*T2*^*/HPIP*^f/f^ mice than in their *HPIP*^f/f^ littermates (Fig. 2d). In addition, we also investigated the skeletal phenotype of *HPIP* complete knockout mice, the *HPIP* complete knockout mice also showed proportional dwarfism than the wild-type littermates (Supplementary Fig. 2b,c). Moreover, the histological assays indicated that within the total limb of *Col2a1-CreER*^*T2*^*/HPIP*^f/f^ mice, the percentage of the proliferative zone was moderately increased, indicating abnormal hypertrophic differentiation caused by *HPIP* insufficiency. The percentage of the hypertrophic zone was also increased while that of the bone zone was decreased in the limbs of *Col2a1-CreER*^*T2*^*/HPIP*^f/f^ mice, suggesting that *HPIP* insufficiency affected not only chondrocyte hypertrophy but also subsequent steps, such as matrix degradation (Fig. 2e and Supplementary Fig. 1c). We then performed immunohistochemical staining, qRT-PCR and western blotting to detect the expression of the cartilage matrix components *COL2A1*, *ACAN*, and *COLX*. The results confirmed that *COL2A1* and *ACAN* expression was suppressed, whereas *COLX* expression was increased in the articular joint of *Col2a1-CreER*^*T2*^*/HPIP*^f/f^ mice compared with that of their *HPIP*^f/f^ littermates (Fig. 2f, g and Supplementary Fig. 1d–f).

### Ablation of *HPIP* prevents OA development

**Fig. 3:**
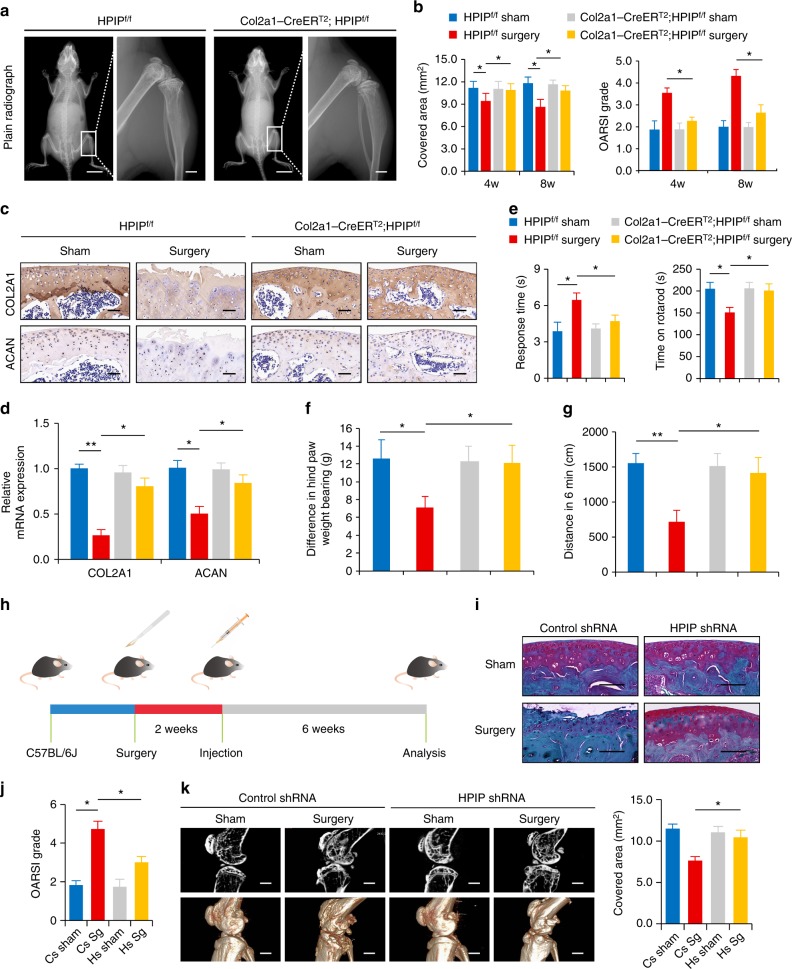
*HPIP* ablation protects mice from the progression of osteoarthritis (OA). **a** Plain radiographs of the entire body (left) and knee (right) of the *HPIP*^f/f^ and *Col2a1-CreER*^*T2*^*;HPIP*^f/f^ littermates (8 weeks old). Scale bar: 10 mm (left), 1 mm (right). **b** Quantitative analysis of the covered surface area and OARSI grade in mice (*n* = 10). **c** Immunohistochemistry assay with the indicated antibodies in mice 8 weeks after ACLT. Scale bar, 100 μm. **d** qRT-PCR examination of *COL2A1* and *ACAN* in *HPIP*^f/f^ and *Col2a1-CreER*^*T2*^*;HPIP*^f/f^ littermate (8 weeks after ACLT). **e** Time of mice on the rotarod 8 weeks after ACLT and the response time of mice in the hotplate analysis (*n* = 10). **f** Difference in hind paw weight bearing (**g**) in mice (8 weeks old) after ACLT (*n* = 10). **g** Traveled distance per 6 min in an open field, in mice after ACLT (*n* = 10). Data are expressed as the means ± standard deviation. **h** Scheme of the OA treatment with adeno-associated virus (AAV) carrying *HPIP*-specific shRNA in mice. **i** Safranin O/fast green staining of whole tibias of mice. Two weeks after surgery, C57/BL6J mice (8 weeks old) were injected intra-articularly with AAV carrying *HPIP*-specific shRNA and analyzed 6 weeks later. (**j**) The OARSI grades of the mice are shown (*n* = 10). Scale bar, 250 μm. Cs Control shRNA; Hs *HPIP* shRNA. Sg Surgery. **k** Sagittal views of μCT and three-dimensional images of the knee joints. Scale bar, 1 mm. Quantitative analysis of the cartilage surface area is shown (*n* = 10). Error bar represents the standard deviation (s.d.) and *P* value was generated by using one-way ANOVA with Tukey’s post hoc test. Cs Control shRNA; Hs *HPIP* shRNA. Sg Surgery. *P < 0.05; **P < 0.01

Next, we investigated the function of *HPIP* in OA pathogenesis. At 8 weeks, plain radiographs and histomorphometric analyses and μCT showed that the size of the knee in *Col2a1-CreER*^*T2*^*/HPIP*^f/f^ had no significant difference with the *HPIP*^f/f^ littermates (Fig. 3a and Supplementary Fig. 3a, b). Because *HPIP* was shown to function as a corepressor of *PBX1* and *PBX1* has a role in osteogenesis^24^, we then detected the expression of *PBX1* and bone characteristics of bone volume fraction, tissue mineral density, connectivity density, trabecular separation, and trabecular thickness in the *Col2a1-CreER*^*T2*^*/HPIP*^f/f^ and *HPIP*^f/f^ littermates to identify whether the phenotype of the *Col2a1-CreER*^*T2*^*/HPIP*^f/f^ mice was in fact due to increased activity of *PBX1*. The results revealed that the expression of *PBX1* was enhanced in *Col2a1-CreER*^*T2*^*/HPIP*^f/f^ mice. Besides, further analysis showed that an increased bone volume fraction, tissue mineral density, connectivity density, and trabecular thickness while a reduction trabecular separation in *Col2a1-CreER*^*T2*^*/HPIP*^f/f^ mice compared with those of *HPIP*^f/f^ littermates (Supplementary Fig. 3c–h). Then, *Col2a1-CreER*^*T2*^*/HPIP*^f/f^ mice and their *HPIP*^f/f^ littermates underwent surgery to perform anterior cruciate ligament transection (ACLT) in their knee joints. The cartilage thickness and bone area covered by cartilage in mice after surgical transection were examined by histopathological analyses and μCT (Supplementary Fig. 3i, j and Supplementary Table 2). Before sham surgery, *Col2a1-CreER*^*T2*^*/HPIP*^f/f^ mice revealed similar articular cartilage thickness and areas compared with their *HPIP*^f/f^ littermates. However, *HPIP*^f/f^ mice showed lower cartilage thickness and covered cartilage areas compared with *Col2a1-CreER*^*T2*^*/HPIP*^f/f^ mice at 4 and 8 weeks after transection (Fig. 3b and Supplementary Table 2). The OARSI histological grading scale of articular cartilage revealed that *Col2a1-CreER*^*T2*^*/HPIP*^f/f^ mice scored lower than their *HPIP*^f/f^ littermates (Fig. 3b). In addition, histopathological analysis showed that *Col2a1-CreER*^*T2*^*/HPIP*^f/f^ mice displayed less severe OA cartilage degradation (Supplementary Fig. 3k). Furthermore, we assessed the structure of tibial subchondral bone using μCT. The result found improvement of trabeculae connectivity and microarchitecture in *Col2a1-CreER*^*T2*^*/HPIP*^f/f^ mice relative to *HPIP*^f/f^ littermates at 4 and 8 weeks after transection, as demonstrated by the normalization of subchondral bone tissue volume and a lower trabecular pattern factor (Supplementary Fig. 3l, m). Besides the immunohistochemical staining assay and qRT-PCR confirmed decreased expression of *COL2A1* and *ACAN* in noncalcified cartilage of *HPIP*^f/f^ mice compared with that of *Col2a1-CreER*^*T2*^*/HPIP*^f/f^ mice (Fig. 3c, d). Moreover, HPIP deficiency significantly reduced mRNA levels of inflammatory markers, such as *IL-1β*, *TNF-α*, *MMP-13*, and *ADAMTS5* according to the qRT-PCR analysis (Supplementary Fig. 3n). The progression of OA is accompanied by secondary clinical symptoms. Rotarod and hotplate analysis and pain measurement showed that *HPIP*^f/f^ mice had an increased hotplate response time and decreased rotarod time, weight bearing and distance traveled, whereas *Col2a1-CreER*^*T2*^*/HPIP*^f/f^ mice with or without transection revealed no significant differences in rotarod or hotplate response times or weight bearing and distance traveled (Fig. 3e–g), thus suggesting *HPIP* ablation prevents OA progression.

### Gene transfer with *HPIP*-specific shRNA treats OA

To further investigate the role of *HPIP* in OA treatment, 8-week-old C57BL/6J wild-type mice underwent ACLT surgery. Two weeks after transection, an intra-articular injection of AAV encoding *HPIP*-specific shRNA was performed (Fig. 3h). The efficiency of AAV encoding *HPIP*-specific shRNA in the joints was evaluated by immunohistochemical staining assay and western blotting and qRT-PCR (Supplementary Fig. 4a–c). Mice that did not receive the injection of *HPIP*-specific shRNA showed more severe OA cartilage degradation, a higher OARSI grade and increased tibial subchondral bone tissue volume and trabecular pattern factor, and a lower cartilage thickness and covered cartilage area compared with mice that received the injection (Fig. 3h–k and Supplementary Fig. 4d–g). Moreover, we also found a decreased expression of *COL2A1* and *ACAN* and increased expression of *IL-1β*, *TNF-α*, *MMP-13*, and *ADAMTS5* in the cartilage of mice that did not receive the *HPIP*-specific shRNA treatment (Supplementary Fig. 4h–j). Taken together, these data demonstrate that *HPIP* inhibition with *HPIP*-specific shRNA protects the joints from OA cartilage impairment.

### RNA-seq analysis of downstream genes regulated by *HPIP*

**Fig. 4:**
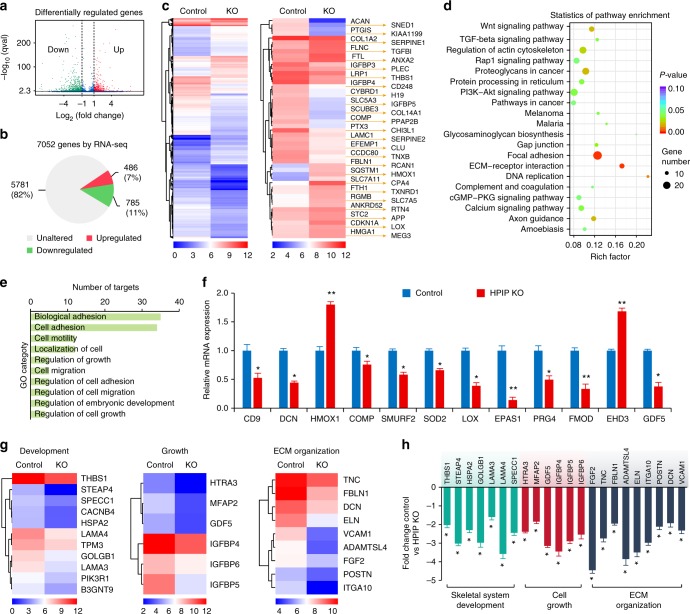
RNA-seq analysis of *HPIP*-modulated genes. **a** A volcano plot illustrating differentially regulated gene expression from RNA-seq analysis between the control and *HPIP* knockout (KO) chondrocytes. Genes upregulated and downregulated are shown in red and green, respectively. Values are presented as the log2 of tag counts. **b** RNA-seq comparison revealed a total of 7052 genes expressed, of which 486 genes were upregulated and 785 genes were downregulated. **c** The hierarchical clustering of the RNA-seq analysis results shows all genes that were significantly different expressed (left panel) and the representative top 50 genes that were differentially expressed (right panel). **d** KEGG pathway analysis of downregulated targets in *HPIP*-deficient transcriptome. **e** gene ontology (GO) functional clustering of genes that were downregulated for biological processes. (The top 10 most significantly affected categories are shown). **f** qRT-PCR validation analysis of the indicated genes regulated by *HPIP*. **g** Heatmap of representative cartilage development, growth, and extracellular matrix (ECM) organization-related genes. **h** qRT-PCR validation analysis shows the mRNA expression fold change of skeletal system development, cell growth and ECM organization-associated genes in the control vs *HPIP* KO chondrocytes. Error bar represents the standard deviation (s.d.) and *P-* value was generated by using one-way ANOVA with Tukey’s post hoc test. *P < 0.05; **P < 0.01

We then examined *HPIP*-mediated transcriptional targets that could account for cartilage degeneration during the pathogenesis of OA. RNA-seq analysis was performed in *HPIP* KO and the control chondrocytes. Before the RNA-seq analysis, the KO efficiency of *HPIP* in chondrocytes was confirmed by western blotting (Supplementary Fig. 5a). Among the 1271 significantly differentially expressed genes (DEGs), transcripts of 486 (7%) genes were upregulated, whereas transcripts of 785 (11%) genes including *COL2A1* (*P* *=* 3.57 × 10^−5^, fold difference = −3.3084) were downregulated in *HPIP* KO compared to the control chondrocytes (Fig. 4a, b). The representative top 50 most significant genes with altered expression are shown (Fig. 4c and Supplementary Fig. 5b–e and Supplementary Tables 3 and 4). In addition, gene ontology (GO) enrichment analysis demonstrated significantly affected categories in genes that were downregulated or upregulated in response to *HPIP* deficiency. The downregulated genes were associated with ECM structural constituents (GO: 0031012, 0044420, 0005578) and the extracellular region (GO: 0005576, 0044421) (Fig. 4e and Supplementary Fig. 6a, b and Supplementary Table 5). Upregulated GO terms indicating amino acid transport (GO: 0003333, 0006865), cell cycle arrest (GO: 0007050) and cell death (GO: 0010941), combined with pathway analysis results of glutathione metabolism and apoptosis (Supplementary Fig. 6c-f and Supplementary Table 6), suggesting that *HPIP* is closely involved in chondrocyte homeostasis. As RNA-seq analysis indicated the altered expression of genes related to cell adhesion which is supported by previous findings that were attributed to *HPIP* functions, we then performed cell adhesion and migration assay to investigate the function of *HPIP*. The results revealed that *HPIP* deficiency inhibited cell adhesion and migration in chondrocytes (Supplementary Fig. 6g, h). Furthermore, KEGG pathway analysis showed that DNA replication, the ECM–receptor interaction and *Wnt* signaling pathways were downregulated, whereas the *p53* signaling pathway was upregulated in *HPIP* KO chondrocytes (Fig. 4d and Supplementary Fig. 6f and Supplementary Tables 7 and 8). Among the 1271 significantly DEGs regulated by *HPIP*, over 400 genes have previously been reported to be associated with OA. We then performed qRT-PCR to examine the representative 12 genes mediated by *HPIP* (Fig. 4f). The results indicated that *HMOX1* and *EHD3* were upregulated in *HPIP* KO chondrocytes, whereas genes such as *CD9*, *DCN*, *SMURF2*, *GDF5*, and so on were downregulated in *HPIP* KO chondrocytes (Fig. 4f). Moreover, many of the DEGs are crucial for development, growth and ECM organization (Fig. 4g). We then validated the differentially altered targets using qRT-PCR. Consistent with the RNA-seq data, qRT-PCR confirmed the mRNA expression of the targets (Fig. 4h). Taken together, these data reveal that *HPIP* acts as a crucial regulator in OA articular cartilage homeostasis.

### ChIP-seq analysis of direct transcriptional targets of *HPIP*

**Fig. 5:**
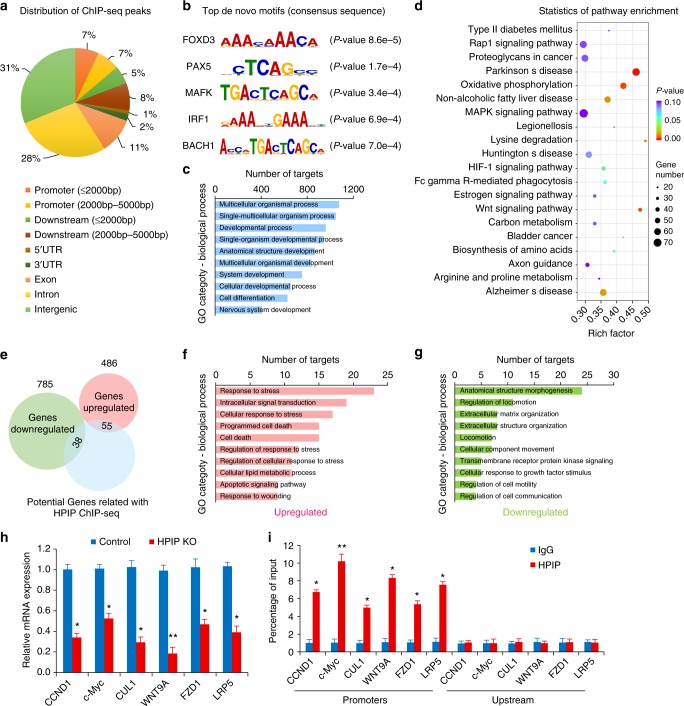
Chromatin immunoprecipitation (ChIP) analysis of cartilage homeostasis-related genes regulated by *HPIP*. **a** *HPIP*-binding regions are mapped by ChIP-seq. The annotation shows that a peak is in the promoter (defined as ≤2000 bp), the promoter (defined as 2000–5000 bp), downstream of the promoter (defined as ≤2000 bp), downstream of the promoter (defined as 2000–5000 bp), 5′ UTR, 3′ UTR, intron or intergenic. **b** Top de novo motifs enriched in the vicinity of the *HPIP*-binding sites. **c** GO functional clustering of targets associated with *HPIP* ChIP-seq peaks for biological process (top 10 most significantly affected categories are shown). **d** KEGG pathway enrichment analysis of targets associated with *HPIP* ChIP-seq peaks. **e** An overlay of RNA-seq and ChIP-seq analysis results revealed genes as potential direct targets of *HPIP* in chondrocytes. **f**, **g** Gene ontology (GO) functional clustering of genes that were upregulated and downregulated for identification of biological processes directly regulated by *HPIP*, respectively (top 10 categories most significantly affected are shown). **h** qRT-PCR validation analysis of the indicated genes in the control vs *HPIP* KO chondrocytes. **i** ChIP assay for *HPIP* occupancy on the *CCND1*, *c-Myc*, *CUL1*, *WNT9A*, *FZD1*, and *LRP5* promoters or upstream of the promoters in chondrocytes. Error bar represents the standard deviation (s.d.) and *P-*value was generated by using one-way ANOVA with Tukey’s post hoc test. *P < 0.05; **P < 0.01

To further explore the underlying molecular mechanisms of *HPIP*-mediated OA cartilage degeneration and assess the direct downstream transcriptional targets of *HPIP*, we performed a genome-wide ChIP-seq assay. The ChIP-seq data were examined by a quality evaluation (Supplementary Fig. 7). The results demonstrated that of 3136 significant ChIP-seq peaks for *HPIP*, including *PBX1* (Fig. 5a and Supplementary Fig. 8a). De novo motif analysis suggested that the most highly enriched DNA-binding motifs among *HPIP*-bound regions occurred in the *FOXD3*, *PAX5*, *MAFK*, *IRF1*, and *BACH1* genes (Fig. 5b), indicating that these transcription factors may act as effector factors of *HPIP*. Besides, GO analysis revealed that these targets were involved in crucial organism processes, cell differentiation and the extracellular matrix (Fig. 5c and Supplementary Fig. 8b, c and Supplementary Table 9). Moreover, KEGG pathway analysis showed potential targets involved in Parkinson’s disease, oxidative phosphorylation and the *Wnt* signaling pathway (Fig. 5d). We then analyzed the intersection of the ChIP-seq and RNA-seq data and found that 93 targets were directly regulated by *HPIP* deficiency (Fig. 5e). GO analysis of the direct targets of *HPIP* deficiency indicated that the upregulated categories included intracellular signal transduction and protein kinase activity (Fig. 5f and Supplementary Fig. 8d, e), whereas the downregulated categories due to *HPIP* deficiency were highly enriched for anatomical structure morphogenesis and extracellular matrix organization (Fig. 5g and Supplementary Fig. 8f, g), which are in line with the results of RNA-seq. We then validated the altered expression of a subset of targets from the intersection of the ChIP-seq and RNA-seq data using qRT-PCR. The results confirmed that the mRNA expression levels of the downstream targets of the *Wnt* signaling pathway (*CCND1*, *c-Myc*, *CUL1*, *WNT9A*, *FZD1,* and *LRP5*) were significantly downregulated in *HPIP*-deficient chondrocytes (Fig. 5h). Furthermore, ChIP analysis found a significant enrichment of *HPIP* in the *Wnt* target gene promoter regions (Fig. 5i). Therefore, we focused on the *Wnt* signaling pathway to decipher the molecular network around *HPIP* in OA cartilage degeneration.

### *HPIP* stimulates *Wnt* signaling by interacting with *LEF1*

**Fig. 6:**
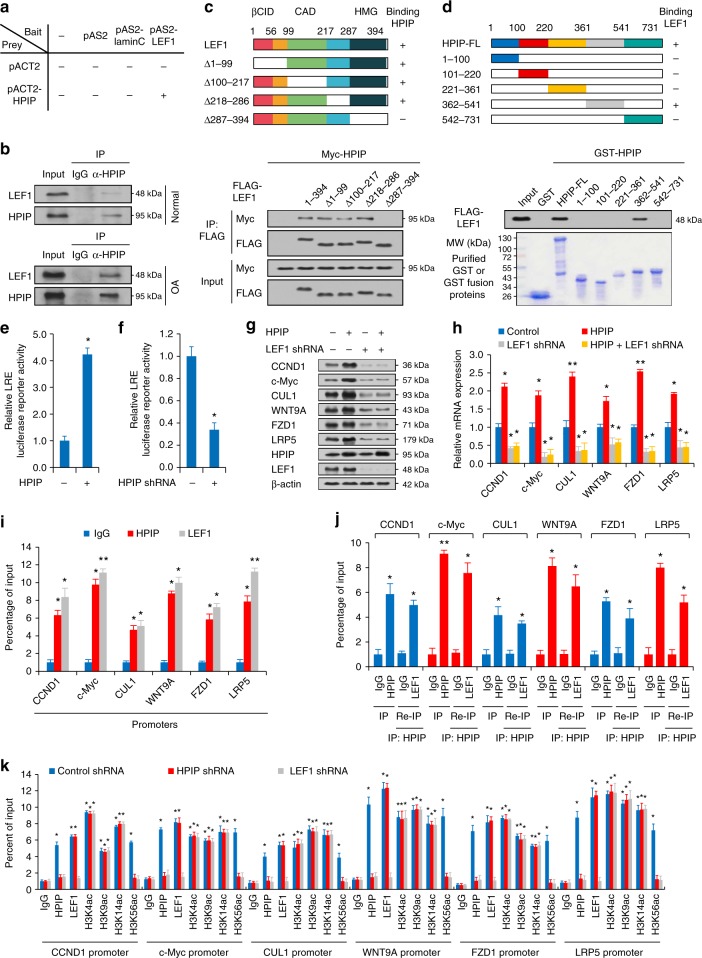
*HPIP* acetylates *H3K56ac* at the promoters of *Wnt* target genes. **a** Yeast CG1945 cells were transformed with the indicated plasmids (bait and prey for the two-hybrid assay) and grown on selective media. Positive interaction is indicative of colonies that grow on selective media and have β-galactosidase activity. **b** Normal or osteoarthritis (OA) chondrocytes were immunoprecipitated with anti-*HPIP* or normal IgG, and the precipitates were analyzed by immunoblot with the indicated antibodies. IP immunoprecipitation. **c** The top panel shows a schematic diagram of the *LEF1* fragments used to map the interaction with *HPIP*. Δ deficient fragment, βCID *β-catenin* interaction domain, CAD context-dependent activation domain, HMG high-mobility group domain. Cell lysates of the *LEF1* fragments were IP with the indicated antibodies. **d** The top panel shows a schematic diagram of the *HPIP* fragments used to map the interaction with *LEF1*. Immunoblot analysis of bound proteins in lysates of OA chondrocytes expressing *FLAG-LEF1* following incubation with Sepharose beads coupled to *GST* alone or various *GST-HPIP* fusion proteins. **e**, **f** Luciferase assay in cultured chondrocytes infected with *LEF1*-response element (LRE) reporter and *HPIP* (**e**) or *HPIP* shRNA (**f**). **g** Cultured human chondrocytes were infected with the HPIP plasmid and the vector control or shRNA for *LEF1*. Immunoblots were incubated with the indicated antibodies. **h** qRT-PCR analysis of the indicated group. **i** ChIP analysis of *HPIP* and *LEF1* occupancy on the indicated *Wnt* target gene promoters. **j** Re-ChIP analysis of the occupancy of *HPIP* and *LEF1* on the indicated *Wnt* target gene promoters. **k** ChIP analysis of *HPIP*, *LEF1*, and histone H3 acetylation occupancy on the indicated *Wnt* target gene promoters in *HPIP* or *LEF1* knockdown chondrocytes. Error bar represents the standard deviation (s.d.) and *P-*value was generated by using one-way ANOVA with Tukey’s post hoc test. *P < 0.05; **P < 0.01

Interactions of transcription factors with genes are closely associated with active transcription. To investigate how *HPIP* regulates *Wnt* target gene transcription, we used the Yeast Two Hybrid System combined with coimmunoprecipitation (Co-IP) to identify its interaction partners. The Yeast Two Hybrid experiment revealed that the transcription factor *LEF1* physically interacted with *HPIP* (Fig. 6a). CO-IP of endogenous proteins confirmed the *HPIP/LEF1* interaction in chondrocytes (Fig. 6b). As *LEF1* was a good partner of *β-catenin*, we performed subcellular separation combined with immunoprecipitation assay to investigate the role of *β-catenin* involved in *HPIP/LEF1* complex. The results revealed that *HPIP* was located in both nucleus (32%) and cytoplasm (68%) in chondrocytes. Endogenous *HPIP* interacted with endogenous *β-catenin*, from both cytoplasmic and nuclear fractions of cells. Endogenous *HPIP* or *β-catenin* associated with endogenous *LEF1* in the nucleus (Supplementary Fig. 9a). Next, we performed the immunofluorescence analysis and found *HPIP* could be colocalized with *LEF1* in the nucleus (Supplementary Fig. 9b). More importantly, *β-catenin* knockdown significantly diminished the physical interaction between *HPIP* and *LEF1*, indicating that the interaction of *HPIP* and *LEF1* is mediated by *β-catenin* (Supplementary Fig. 9c). We then examined the region of *LEF1* that interacts with *HPIP* using synthesized *LEF1* deletions and a CO-IP assay (Fig. 6c). All of the deletions from the N terminus of *LEF-1* (Δ1–99, Δ100–217 and Δ218–286) retained the ability to interact with *HPIP*, whereas removal of the high mobility group (HMG) domain of *LEF1* (Δ287–394) eliminated the interaction with *HPIP* (Fig. 6c), suggesting that the interactions are localized to the *LEF-1* HMG domain.

Next, we performed GST pull-down experiments to identify the region of *HPIP* that interacts with *LEF1* (Fig. 6d). The results showed that the FLAG-tagged *LEF1* protein interacted with purified GST-*HPIP*, but not GST alone (Fig. 6d). Besides, we noticed that the molecular weight of *HPIP* (1–100) was little larger than that was predicted by the sequence data, which might be caused by posttranslational modifications because its coding sequence and open reading frame was correct according to the DNA sequencing results. Importantly, we found that *HPIP* (362–541), but not other *HPIP* regions (1–100, 101–200, 221–361, 542–731), was associated with *HPIP* (Fig. 6d), indicating that region 362–541 is necessary for the *HPIP–LEF1* interaction.

### *HPIP* acetylates *H3K56ac* at the promoters of *Wnt* target genes

Next, we examined how *HPIP* regulates *Wnt* target gene transcription in OA. We first performed a luciferase reporter assay that revealed that the luciferase activity of *LEF1*-response element (LRE) was significantly increased by introduction of *HPIP* into chondrocytes (Fig. 6e), whereas inhibition of *HPIP* diminished this activity (Fig. 6f), suggesting that *HPIP* might promote *Wnt* signaling activation through *LEF1*. We then examined whether *LEF-1* is required for *HPIP*-mediated expression of *Wnt* target genes. The results showed that *HPIP* overexpression increased the expression of *Wnt* targets (*CCND1*, *c-Myc*, *CUL1*, *WNT9A*, *FZD1,* and *LRP5*), whereas knockdown of endogenous *LEF-1* inhibited the expression of *Wnt* targets (*CCND1*, *c-Myc*, *CUL1*, *WNT9A*, *FZD1,* and *LRP5*) and abolished the ability of *HPIP* to modulate *Wnt* target production (Fig. 6g, h), indicating that *LEF1* is important for *HPIP*-mediated *Wnt* target gene expression regulation.

Furthermore, the ChIP assay revealed that like *HPIP*, *LEF1* was recruited to the *HPIP*-binding sites of the *CCND1*, *c-Myc*, *CUL1*, *WNT9A*, *FZD1*, and *LRP5* promoters in chondrocytes (Fig. 6i). Re-ChIP experiments showed that *HPIP* was associated with *LEF1* on the corresponding binding sites (Fig. 6j). Because the histone acetylation regulated by histone acetyltransferases is often associated with active transcription of genes, we then investigated whether *HPIP* regulates *Wnt* target expression via modification of the chromatin status in chondrocytes. *LEF1* knockdown reduced the recruitment of *HPIP* and acetylation of *H3K56ac*, but not *H3K4ac*, *H3K9ac* or *H3K14ac*, to the promoters of *CCND1*, *c-Myc*, *CUL1*, *WNT9A*, *FZD1,* and *LRP5* in chondrocytes (Fig. 6k). *HPIP* inhibition also caused marked suppression of recruitment of *H3K56ac*, but not *H3K4ac*, *H3K9ac*, and *H3K14ac*, to the *HPIP/LEF1* binding sites, indicating that *H3K56ac* activity is important for *HPIP* modulation of gene transcription. Site specificity of histone is mainly determined by distinct types of histone acetyltransferases (HATs) complex. To further investigate how *HPIP/LEF1* complex specifically promotes *H3K56ac*, we searched for the potential HATs in *HPIP*-preying proteins screened out by Yeast Two Hybrid assay and found two HATs, *p300* and *GCN5* as the potential *HPIP/LEF1* partners. Co-IP assay further confirmed that acetyltransferase *p300*, acetylating *H3K56* site, rather than *GCN5*^25^,, acetylating *H3K4*, *H3K9*, and *H3K14* sites, specifically interacted with *HPIP*, which might explain for the site acetylation preference of the histone (Supplementary Fig. 9d, e). We next evaluated the proliferative activities of *LEF1* and *HPIP* in chondrocytes using cell growth assays. In line with the above results, *HPIP* or *LEF1* inhibition suppressed the proliferation of chondrocytes (Supplementary Fig. 9f, g)

The findings above have shown that *H3K56ac* was involved in gene expression and there were enhanced *H3K56ac* around *HPIP* DNA-binding regions. *H3K56ac* was shown as a histone marks observed preferably during stress response at the replication forks, which was in line with our RNA-seq data from KO cartilage that showed cell death, DNA replication, and *p53* pathway were significantly altered (Supplementary Tables 6–8), we then tested the possible connection of *HPIP* KO-*H3K56ac* in DNA replication and stresses response and its role in OA progression. The results indicated that *HPIP* KO diminished the recruitment ability of *H3K56ac* to the promoters of DNA replication and stresses response genes, which were derived from RNA-seq data (Supplementary Fig. 10a–f). As *HPIP* is overexpressed in OA and *HPIP* causes increased *H3K56* status in human OA chondrocytes, we next examined the relationship among the expression of *H3K56ac*, *HPIP* and *LEF1*. The results showed that the level of *HPIP* expression was positively correlated with *H3K56ac* or *LEF1* protein expression. Besides, *H3K56ac* expression was also positively associated with *LEF1* protein expression (Supplementary Fig. 10g–k). Taken together, these data demonstrate that *HPIP* directly activates expression of key *Wnt* signaling pathway targets by acetylating *H3K56ac* in their promoters in OA and *HPIP* had a crucial role in the OA progression.

## Discussion

In the present study, we identified increased *HPIP* expression levels during OA progression. *HPIP* deficiency in mice impairs articular cartilage development and prevents the development of OA. Importantly, an intra-articular injection of AAV carrying a *HPIP*-specific shRNA showed protective effects against OA. The molecular network around *HPIP* was demonstrated at the molecular, cell, histological, functional, and clinical levels. Mechanistically, we found that *HPIP* physically binds to *Wnt* signaling transcriptional factors (*LEF1*). Furthermore, *HPIP* colocalizes with *LEF1* and activates the transcriptional activity of *LEF1* and acetylates *H3K56ac* in the promoters of *Wnt* signaling pathway targets to modulate OA pathogenesis and *HPIP* expression is positively correlated with *H3K56ac* or *LEF1* protein expression, suggesting that *HPIP* might be a promising therapeutic target against OA.

*HPIP* was originally identified in a yeast two-hybrid screen as a *PBX1*-binding protein^26^, and was shown to function as a corepressor of this transcription factor. *HPIP* participates in proliferation and progression. Our previous study revealed that *HPIP* regulates HBV X protein (HBx)-expressing hepatocarcinoma cell growth, invasion, EMT and metastasis through the *AKT/mTOR* or *ERK/mTOR* pathway^16^. In addition, *HPIP* interacts with casein kinase 1α (*CK1*α) and predicts the clinical outcomes for renal carcinoma^27^. *HPIP* also facilitates gastric cancer cell proliferation through activation of G1/S and G2/M cell cycle transitions and modulates colorectal cancer progression through activation of the *MAPK/ERK1/2* and *PI3k/AKT* pathways^17,28^. Moreover, *HPIP* upregulation has been found in lung cancer^29,30^, ovarian cancer^31–33^, breast cancer^18,34^, thyroid cancer^35^, spinal glioblastoma^36^, head-and-neck squamous cell carcinoma^37^, and oral squamous cell carcinoma^38^, which indicates that *HPIP* is an important essential mediator in tumorigenesis. However, the exact role of *HPIP* in OA pathogenesis remains unknown. In the present study, we found that *HPIP* expression was significantly higher in OA cartilage. *HPIP* promoted the cell proliferation in chondrocytes. To characterize the specific functions of *HPIP* in cartilage development, we generated the chondrocyte-specific *HPIP* KO mice (*Col2a1-CreER*^*T2*^*/HPIP*^f/f^ mice) and the mice revealed defects in bone development and proportional dwarfism compared to wild-type littermates. Similar observations that the cartilage specific cKO mice affect bone formation could also be found in other previous studies, such as *Kdm6b* cKO^15^, *Rac* cKO and *Cdc42* cKO mice^39^. We speculated that the phenomenon might be related to the following reasons. First, the skeleton is mostly formed through the process of cartilage replacement in the growth plates of bones in a series of distinct chondrocyte differentiations steps, therefore, cartilage defects affect bone formation. Second, RNA-seq analysis suggested that *HPIP* could regulate a set of makers associated with ECM organization and skeletal system development. Disorganized structure of ECM also affects abnormal skeletal system development. In terms of *PBX1*, as a transcription factor, *PBX1* possesses the characteristics of activation or repression regulatory ability. Gordon et al.^24^ reported that *PBX1* repressed osteoblastogenesis by blocking *Hoxa10*-mediated recruitment of chromatin remodeling factors. However, Selleri et al.^26^ reported that *PBX1* homozygous knockout mice revealed several defects in skeletogenesis, suggesting that *PBX1* is required for bone formation and skeletal patterning. Moreover, Cheung et al.^40^ reported that silencing of *PBX1* decreased expression of *Runx2* and *Osterix*, the two indispensable factors required for osteogenesis, indicating *PBX1* has the cooperative function in osteogenesis. *PBX1* protein is unable to activate or repress transcription alone^41^, but has to bind to other partner proteins including histone modification enzymes to exhibit the transcriptional function. The activation or repression function of *PBX1* can be dependent on the type of histone modification enzymes it binds to and the type of bone-related gene promoters it recruits to. In the present study, we found *PBX1* regulated osteogenesis. The detailed mechanisms underlying the *PBX1*-mediated regulation in osteogenesis remain to be further elucidated.

To further assess the structure of tibial subchondral bone, we performed the μCT and histology assay. Our histology results and bone tissue volume data obtained from μCT demonstrated that the subchondral bone plate (SBP) thickened in response to OA and cKO of *HPIP* led to decreased subchondral plate thickness and volume after surgery. The subchondral bone tissue volume of μCT data was in line with the histology findings, which is consistent with what we have expected. Moreover, we reported that the *HPIP* ablation genetic model could protect against developing OA. According to the combination of μCT and histopathological analysis, an intra-articular injection of AAV carrying *HPIP*-specific shRNA in C57BL/6J wild-type mice after ACLT surgery showed a protective effect against OA cartilage degradation, which supports *HPIP*’s role as a potential clinical therapeutic candidate for OA treatment.

To investigate the molecular mechanisms underlying the function of *HPIP* in OA, we performed RNA-Seq analysis to search for *HPIP*-mediated downstream target genes. We found a total of 1271 DEGs between the control and *HPIP* KO chondrocytes, including over 400 previously reported OA-associated genes^19–21,42–50^, which suggests that *HPIP* is closely related to the pathogenesis of OA. Moreover, *HPIP*-deficient chondrocytes were also found to exhibit decreased expression of *CD9*, *DCN*, *EPAS1*, *FMOD*, and *SMURF2*, which have been reported to be inducers of OA. *CD9* deficiency mice show a reduced severity of hallmarks of OA, including less cartilage degradation and soft tissue inflammation^19^. *DCN*-deficient mice develop significantly less OA after forced exercise than wild-type mice^20^. *EPAS1*-deficient mice show resistance to OA development^46^. The *Bgn/FMOD* double-deficient mouse demonstrates an increase in bone and subchondral bone volume as well as in the expression of collagen type X and aggrecan compared with wild-type controls^48^. *SMURF2* has been reported to cause spontaneous OA in mice^43^. Our study showed that *HPIP* is as an upstream regulator of these five known critical OA inducers (*CD9*, *DCN*, *EPAS1*, *FMOD*, and *SMURF2*), extending our understanding of the importance of *HPIP* in OA regulation. Moreover, we identified that *HPIP*-deficient mice protect against developing OA. Importantly, the intersection of ChIP-seq and RNA-seq data indicated that HPIP-modulated OA pathogenesis through regulation of *Wnt* signaling.

The *Wnt* signal transduction pathway is an important main regulator of development and disease^51–53^. The *Wnt* signaling pathway controls normal aging and contributes to human OA^54–56^. In this study, we found that *HPIP* deficiency in OA chondrocytes impaired *Wnt* signaling. Subsequent analysis demonstrates that *HPIP* exerts its biological function mainly through interactions with transcriptional factors (*LEF1*) to activate *Wnt* signaling. *LEF1* is a sequence-specific DNA-binding protein which is expressed in adult mice pre-B and T lymphocytes and in the mesencephalon, neural crest, whisker follicles, tooth germs, and other sites during the progression of embryogenesis^57,58^. Previous reports have shown that *LEF1*-null mice exhibit developmental defects in teeth, hair follicles, mammary glands, and the brain^58^. Besides, in terms of skeletal metabolism, analysis of *LEF1*-null mice is hampered by their perinatal lethality^59^. In the present study, we also found *LEF1* knockdown inhibited the proliferation of chondrocytes, which was in line with the previous findings. *HPIP* inhibition results in marked suppression of recruitment of *H3K56ac* to the promoters of *Wnt* target genes. *H3K56ac* was shown to be a histone marks observed preferably during stress response at the replication forks, which was in line with our RNA-seq data from KO cartilage that showed cell death, DNA replication, and *p53* pathway were significantly altered. In the present study, we also found *HPIP* KO diminished the recruitment ability of *H3K56ac* to the promoters of DNA replication and stresses response genes. Besides, *H3K56ac* expression was also positively correlated with *HPIP* and *LEF1* protein expression, revealing a molecular network around *HPIP*, *LEF1*, and *H3K56ac* dynamics and *Wnt* signaling in OA.

Collectively, our findings demonstrated that *HPIP* expression was increased in OA. *HPIP* deficiency in mice impairs articular cartilage development and protects against developing OA. An intra-articular injection of AAV carrying *HPIP*-shRNA in vivo attenuated OA articular cartilage degradation when administered after injury. Mechanistically, we showed that *HPIP* physically interacts with *LEF1* to promote transcription of *Wnt* target genes. *HPIP* potentiates *LEF1* transcriptional activity and acetylates *H3K56ac* around *Wnt* signaling target gene promoters, thus suggesting that *HPIP* may be an attractive therapeutic target for treating OA patients. Further investigation is warranted to evaluate the efficacy of lentivirus encoding *HPIP*-specific shRNA in OA models of large animals before the clinical trials.

## Methods

### Patients and specimens

All human studies were conducted with informed consent of the patients and approval of the Institutional Ethics Review Board of the General Hospital of the People’s Liberation Army (Beijing, China). OA was macroscopically diagnosed according to the Modified Outerbridge Classification^60,61^ (stage 0, intact cartilage; stage 1, chondral softening and or blistering with intact surface; stage 2, superficial ulceration, fibrillation, or fissuring < 50% of depth of cartilage; stage 3, deep ulceration, fibrillation, fissuring, or chondral flap > 50% of cartilage without exposed bone; and stage 4, full-thickness wear with exposed subchondral bone). Articular cartilage samples were collected from 118 patients with knee OA who underwent knee arthroplasty surgery. Specimens that included all cartilage layers and subchondral bone were separately harvested from sites on the tibial plateau by drilling holes (4.5 mm). Clinical information was collected from patient records. The clinical and demographic characteristics of the study population are shown (Supplementary Table 1).

### Mice

*HPIP*^f/f^ mice were generated by Cyagen Biosciences Inc. *Col2a1-CreER*^*T2*^ mice (Stock Number: 006774) were obtained from Jackson Laboratories (Bar Harbor, ME, USA). Both *HPIP*^f/f^ and *Col2a1-CreER*^*T2*^ mice were in a C57BL/6J background. To generate *Col2a1-CreER*^*T2*^*; HPIP*^f/f^ mice, *HPIP*^f/f^ mice were mated with *Col2a1-CreER*^*T2*^ mice to obtain *Col2a1-CreER*^*T2*^*; HPIP*^f/+^ mice, which were then mated with *HPIP*^f/f^ mice. *HPIP* general knockout mice were generated by Shanghai Model Organisms Center, Inc using CRISPR/Cas9. The guide RNA targeting exon 3 of *HPIP* gene was designed. The guide RNA sequence is TGGAGGAGTCCCGAAGGGCCTGG. The exon 3 region in HPIP is from 225 to 247. A mixture of plasmids encoding *Cas9* and *HPIP* guide RNA was microinjected into the fertilized C57BL/6 eggs, and the transgenic embryos were planted into pseudopregnant recipients. Founder lines of successful mutation of the *HPIP* gene cluster were identified through PCR genotyping of tail DNA. The positive female founder mice and wild-type male mice were bred to get F1 *HPIP* heterozygote mice. Male *HPIP* heterozygote mice and female *HPIP* heterozygote mice were then crossed to obtain *HPIP* homozygote mice. For mouse embryo genotype identification, genomic DNA was prepared from the tail tips of 14-day-old embryos and the *HPIP* mutation was identified by PCR amplification, DNA sequencing and immunoblot. All mice used in the OA evaluation were males to avoid any potential postmenopausal bone loss effect and were maintained under pathogen-free conditions. All experimental procedures were approved by the Institutional Animal Care and Research Advisory Committee of the General Hospital of the People’s Liberation Army.

### ACLT surgery

Eight-week-old *Col2a1-CreER*^*T2*^*; HPIP*^f/f^ mice and their *HPIP*^f/f^ littermates were injected intraperitoneally with tamoxifen (Sigma, St. Louis, MO, USA; 100 μg/g body weight) daily for 5 days before surgical induction of OA. We anesthetized the mice from this group with ketamine and xylazine and then underwent ACLT surgical transection of the right knee to induce mechanical instability and create an experimental OA model. Sham operations were performed on control mice. We analyzed *Col2a1-CreER*^*T2*^*; HPIP*^f/f^ mice and their *HPIP*^f/f^ littermates 4 and 8 weeks after surgery. Investigators were blinded to the genotype of the mice when surgical transection was performed. The OARSI system (grades 0–6) was used to quantify OA severity, which was also evaluated by observers who were blinded to the experimental group.

### Cell culture

Normal chondrocytes were obtained from the knees of patients who had died of diseases unrelated to arthritis. Arthritic chondrocytes were obtained from patients undergoing elective total knee arthroplasty for end-stage OA according to a modified Outerbridge scale (Grade III: maximal fibrillation). The chondrocytes were incubated in high glucose Dulbecco’s modified Eagle’s medium (DMEM) with 10% fetal calf serum (FCS), 100 IU/ml penicillin and 100 μg/ml streptomycin at 37 °C in an atmosphere of 5% CO_2_. The characterization of the chondrocyte phenotype was established through the analysis of *COL2/COL1* gene expression. First-passage chondrocytes at 85% confluence were used for all experiments.

### *HPIP* knockout chondrocytes

*HPIP* knockout (KO) chondrocytes were generated by *CRISPR/Cas9* (Santa Cruz Biotechnology, Inc.). The *CRISPR/Cas9* KO Plasmid (sc-412788) and the control *CRISPR/Cas9* Plasmid (sc-418922) infection for HPIP were performed according to the manufacturer’s instructions. Briefly, the Plasmid DNA solution was added directly to the dilute UltraCruz® Transfection Reagent (sc-395739) using a pipette, and incubated for 30 min at room temperature. Then the chondrocytes were incubated for 24–72 h under conditions normally used to culture the cells. The KO efficiency of *HPIP* in chondrocytes was then confirmed by western blotting.

### RNA-sequencing analysis

Isolated *HPIP* KO and the control chondrocytes were filtered through 70 mM nylon filters (BD), washed twice with sterile PBS, and then were directly prepared for cDNAs amplification and RNA-Seq library construction without any culture. Total RNA was isolated with TRIzol reagent from *HPIP* KO and the control chondrocytes. A complementary DNA library was prepared and sequencing was performed according to the Illumina standard protocol by Beijing Novel Bioinformatics Co., Ltd. (https://en.novogene.com/). Raw reads from RNA-seq libraries were trimmed to remove the adaptor sequence and the reads with adaptor contaminants and low-quality reads (the mass value *Q*-score < 5 of the base number accounts for more than 50%) and reads from *N* (*N* indicates that the base information that cannot be determined) which is >10%. After filtering, reference genome and gene model annotation files were downloaded from a genome website browser (NCBI/UCSC/Ensembl). Indexes of the reference genome were built using Bowtie v2.0.6 and paired-end clean reads were aligned to the reference genome using TopHat v2.0.9. Bowtie was used for a BWT (Burrows–Wheeler Transformer) algorithm for mapping reads to the genome and Tophat can generate a database of splice junctions based on the gene model annotation file and thus achieve a better mapping result than other non-splice mapping tools. For the quantification of gene expression level, HTSeq V0.6.1 was used to count the read numbers mapped for each gene. The RPKM of each gene was calculated based on the gene read counts mapped to this gene. A differential expression analysis was performed using the DESeq R package (1.10.1). For clustering, we clustered different samples to see the correlation using hierarchical clustering distance method with the function of heatmap, SOM (Self-organization mapping) and kmeans using silhouette coefficient to adapt the optimal classification with default parameter in R. The RNA-seq data in this study was deposited at Gene Expression Omnibus (GEO) (http://www.ncbi.nlm.nih.gov/geo/) under accession ID GSE100312.

### ChIP-sequencing assay

A ChIP assay was performed using a commercially available kit (Millipore, MA, USA)^62^. One hundred nanograms of DNA fragments were used per ChIP. Sequencing libraries were prepared from collected *HPIP* ChIP and corresponding input DNA by blunting, A-tailing and adaptor ligation using NEXTFlex barcoded adapters (Bioo Scientific). Libraries were PCR-amplified for 16 cycles, fragments with size of 300 bp were processed using the Covaris Focused-ultrasonicators (Covaris, Inc. S220). Sequencing was performed using the Hi-Seq 2500 (Illumina) platform at Beijing Novel Bioinformatics Co., Ltd. (https://en.novogene.com/). ChIP-seq data were aligned to the hg19 genomes using bowtie-0.12.9. The basic quality statistics analysis of raw reads was evaluated by the FastQC software. Raw reads were then trimmed to remove the duplicate reads and reads that were matched with the adaptor sequence and the base whose mass value of the 3′-tail base number that was <20. If the remaining sequence length was not shorter than 18 nt, it will be retained. Only tags mapped uniquely to the genome were considered for further analysis. We select 13 as the threshold for mapping quality (MAPQ) to ensure the high quality. Genomic binding peaks for *HPIP* were identified using the findPeaks command from HOMER (http://homer.salk.edu/homer/) with eightfold enrichment over the input sample. Peaks were annotated using the annotatePeaks command. *HPIP*-related transcription factor binding motifs were performed with command findMotifsGenome.pl. The complete ChIP-seq datasets in this study was deposited at GEO database (http://www.ncbi.nlm.nih.gov/geo/) under accession ID GSE100312.

### Gene ontology (GO) and KEGG enrichment analysis

GO enrichment analysis of differentially expressed genes was implemented by the GOseq R Package, in which gene length bias was corrected. GO analysis was performed with the DAVID online tool (https://david.ncifcrf.gov/). Top GO categories were selected according to the *P*-values. Pathways of differentially expressed genes were analyzed by the KEGG database (http://www.kegg.jp/kegg/). We used KOBAS software to test the statistical enrichment of differential expression genes in KEGG pathways.

### Adeno-associated virus (AAV)

The AAV vector was generated after cloning short hairpin RNA (shRNA) fragments into the adeno-associated virus vector GV478 (Shanghai Genechem Co., Ltd). AAV packaging was performed by cotransfecting AAV-293 cells with the recombinant AAV vector, pAAV-RC vector, and pHelper vector. AAV were collected from the AAV-293 cell supernatant, condensed, and purified for further animal experiments.

### Intra-articular administration

C57BL/6J wild-type mice were anesthetized with 3% isoflurane and maintained with a 2% isoflurane and oxygen mixture, and then, the skin above the articular joint was shaved. Adeno-associated virus carrying *HPIP*-specific shRNA was produced as described above. Virus-containing medium was collected and passed through a 0.45 μm filter (Millipore) to remove cell debris. Then, the adeno-associated virus of the supernatant was concentrated by ultracentrifugation at 28,800 × *g* at 4 °C for 2 h. The supernatant was subsequently discarded, and the precipitate was resuspended in 100 ml PBS. Mice were injected intra-articularly with this solution using 33-gauge needles (Hamilton Company) and 25 µl CASTIGHT syringes (Hamilton Company).

### Rotarod and hotplate analysis

Unanesthetized mice were put onto an accelerating rotarod (Ugo Basile). Briefly, the first round time was recognized by the duration to stay atop the rod for the first failure. Mice at different time points after surgery were randomly assigned into different groups. Each trial had a maximum time of 5 min. Mice were given an intertribal rest interval of 30 min. For hotplate analysis, unanesthetized mice of different groups were put on the 55 °C hotplate (Columbus Instruments). Time of response (jumping, licking, or jumping) for the latency period was recorded. Rotarod and hotplate analysis were evaluated by observers who were blinded to the experimental group.

### Pain measurement

Pain behaviors were assessed as hind limb weight-bearing asymmetry. Weight-bearing asymmetry was measured as the difference between hind limbs as a percentage of total weight borne through both hind limbs^63,64^. Mice were acclimated to the test room for 30 min before open field testing^65^. Mice were placed in the center of individual plexiglass square chambers (45 cm × 45 cm) and allowed to freely explore the chamber for the duration of the 6-min test session. The movements of the mice were recorded with a video camera. Upon completion of the test, which was performed once per animal, each mouse was returned to its home cage. Two observers blinded to treatment group assignments manually traced mouse movements to calculate the distance (in cm) that the mouse traveled within the cage in 6 min (traveled distance).

### μCT analysis

Mouse knee joints were harvested, and soft tissues including muscles and skins were dissected. The remaining tissues were including the whole original mouse knee joints were fixed in formaldehyde and then were stored in 70% ethanol. CT scanning was performed using high resolution μCT (Skyscan 1172). Images were analyzed and reconstructed with CTAn v1.9 and NRecon v1.6. Three-dimensional model visualization software (CTVol v2.0) was also used. A voltage of 50 kVp, a resolution of 5.7μm per pixel and a current of 200 μA were set for the scanner. The transverse, coronal and sagittal images of the knee joints were used for analyses. The region of interest covering the surface area of tibia was collected. After scanning, each sample was assigned a random number for ensure blinded assessment, and then was processed for the image processing. The transverse, coronal and sagittal images of the knee joints were used for analyses. The region of interest covering the surface area of tibia was collected. After scanning, each sample was assigned a random number for ensure blinded assessment, and then was processed for the image processing.

### Histology and immunohistochemical assay

Cartilage tissues were fixed in 4% buffered paraformaldehyde for 48 h and subsequently decalcified with buffered EDTA (20% EDTA, pH 7.4). The tissues were embedded in paraffin, sectioned and stained with hematoxylin–eosin (HE), safranin O/fast green and Masson’s trichrome. The thickness of the medial subchondral bone plate (region between the osteochondral junction and marrow space on the medial side of the tibial plateau, in μm) was measured in a blinded fashion with OsteoMeasureXP Software (OsteoMetrics, Inc.). The histopathological changes were blindly scored using the modified Mankin grading system. For whole skeleton staining, mice were collected, and the soft tissues of skin, viscera, and adipose tissue were removed and then fixed in 95% (v/v) ethanol for 1 week. The specimens were then transferred to acetone and stained in a solution containing Alizarin red S and Alcian blue 8GX (Sigma-Aldrich) at 37 °C, protected from light. The rinse the mice in 1% aqueous KOH for 2 days. The specimens were stored in 100% glycerol. Immunohistochemical assay was performed using a standard protocol. Briefly, the articular cartilage sections were pre-treated for 10 min with trypsin (0.05%) prior to treatment with 3% (vol/vol) H_2_O_2_ for 15 min. Subsequently, the sections were blocked with 10% goat serum for 1 h at room temperature. After washing with PBS, primary antibodies (anti-Collagen II, 1:100, Abcam, ab34712; anti-Aggrecan, 1:50, Abcam, ab36861) were applied to the sections and incubated overnight at 4 °C. The sections were subsequently washed with PBS and incubated for 15 min with biotinylated secondary antibody, followed by incubation for 30 min using the Histostain Plus kit (Invitrogen). Lastly, the sections were washed and incubated for 2 min with 3, 3′-diaminobenzidine (DAB) substrate. Using light microscopy, two experienced pathologists blindly reviewed the stained tissue sections. The widely accepted German semi-quantitative scoring system^66^, considering the staining intensity and area extent: 0, negative staining; 1, weakly positive staining; 2, moderately positive staining; and 3, strongly positive staining; The extent of marker expression was quantified by evaluating the percentage of the positive staining areas in relation to the whole area in the core. In addition, a score of 0 (<5%), 1 (5–25%), 2 (25–50%), 3 (51–75%), and 4 (>75%) were given for the reactivity. The final immunoreactive score was determined by multiplying the intensity and the extent scores, yielding a range from 0 to 12. For the assay, we defined a 0 score as negative and scores of 1-12 as positive.

### Plasmids

For the eukaryotic expression vectors encoding *FLAG*-, *MYC*-fusion proteins tagged at the amino terminus were constructed by inserting PCR-amplified fragments into *pcDNA3* (Invitrogen) or *pIRESpuro2* (Clontech). *LEF1*-response element (LRE) promoter luciferase reporters were generated through insertion of PCR-amplified promoter fragments from genomic DNA into the *pGL4*-basic vector (Promega). Lentiviral shRNA vectors were generated after cloning short hairpin RNA (shRNA) fragments into the lentiviral vector *pSIH-H1-Puro* (System Biosciences). Lentiviruses were produced through cotransfection of HEK293T cells (RRID:CVCL_0063) with recombinant lentivirus vectors and the pPACK Packaging Plasmid Mix (System Biosciences) using a Megatran reagent (Origene). Lentiviruses were collected at 48 h after transfection and added to the medium of target cells with 8 μg/ml Polybrene (Sigma-Aldrich). Plasmids encoding GST-fusion proteins were prepared by cloning into *pGEX-KG* (Amersham Pharmacia Biotech). For the yeast two-hybrid assay, the bait plasmid *pAS2-LEF1* was constructed by inserting the full-length *LEF1* cDNA fragment into *pAS2-1* (Clontech). Details of the cloning are available upon request.

### Quantitative reverse-transcription PCR (qRT-PCR)

Total RNA was extracted and reverse-transcribed into cDNA using a RNeasy Mini kit (Qiagen) according to the manufacturer’s instructions. Expression of mRNAs was determined using SYBR Premix Ex Taq Master Mix (2×) (Takara). The relative expression level of the target was calculated using the comparative Ct method. *β-actin* was used as an internal control to normalize sample differences. The sequences of the primers used for qRT-PCR analysis are presented (Supplementary Table 10).

### Western blotting

Total proteins were extracted from tissues or chondrocytes using RIPA buffer (50 mM Tris–HCl (pH 7.4), 150 mM NaCl, 20 mM EDTA, 1% Triton X-100, 1% sodium deoxycholate, 1% SDS and protease inhibitors, Wheaton Science) on ice for 30 min and analyzed using SDS-PAGE electrophoresis. Membranes were incubated with anti-*HPIP* (1:50, Proteintech, 12102-1-AP), anti-*LEF1* (Abcam, 1:200, ab137872), anti-*CCND1* (Abcam, 1:2 000, ab134175), anti-*c-Myc* (Abcam, 1:1 000, ab32072), anti-*CUL1* (Abcam, 1:200, ab75817), anti-*WNT9A* (Abcam, 1:100, ab125957), anti-*FZD1* (Abcam, 1:50, ab71342), anti-*LRP5* (Abcam, 1:50, ab36121). Immunocomplexes were visualized through chemiluminescence using an ECL kit (Amersham Biosciences). Uncropped scans of the most important immunoblots are shown in Supplementary Information (Supplementary Fig. 11).

### Cell adhesion assay

For the adhesion assay, a 96-well plate was coated with fibronectin (Sigma) as the manufacturers’ instructions. Wells were blocked for 30 min with 0.5% heat-inactivated BSA/PBS, rinsed three times with PBS. *HPIP* KO and the control chondrocytes were seeded and incubated at 37 °C for 40 min. After washing with serum-free media, adherent cells were fixed, stained and solubilized for absorbance measurement (650 nm).

### Migration and cell growth assays

Cells were seeded in 6-well plates at 70% confluence in culture medium for migration assays^67^. After 24 h, the confluent cellular monolayer was scratched with a fine pipette tip. For migration, the rate for migration was observed at the indicated times using a microscope. For cell growth, cell proliferation was assessed by using a CCK-8 Kit (Dojindo Laboratories, Kumamoto, Japan) according to the manufacturer’s instructions. Chondrocytes were seeded in 96-well plates and examined at 0, 24, 48, 72, and 96 h.

### Yeast two-hybrid assay

The bait plasmid pAS2-LEF1 and a human mammary two-hybrid cDNA library (Clontech) were sequentially transformed into *Saccharomyces cerevisiae* strain CG1945 according to the manufacturer’s instructions (Clontech). Transformants were grown on synthetic medium lacking tryptophan, leucine, and histidine but containing 1 mM 3-aminotriazole. Approximately one million transformants were screened. The candidate clones were rescued from yeast cells and re-transformed into the same yeast strain to verify the interaction between the candidates and bait. The specificity of the interaction was examined by comparing the interactions between the candidates and various bait constructs. The unrelated bait plasmid pAS2-lamin C was used as a negative control.

### Subcellular fractionation

The localization of proteins was examined by subcellular fractionation. Briefly, cells were homogenized using a Dounce homogenizer, and the homogenate was centrifuged at 366 × *g* for 10 min. The pellet was then analyzed as the nuclear fraction. The supernatant was centrifuged again at −16,200 × *g* for 10 min, and the final supernatant was analyzed as the cytoplasmic fraction.

### Chromatin immunoprecipitation (ChIP) assay and re-ChIP

ChIP assay was performed using the Magna ChIP Assay Kit (Millipore) according to the manufacturer’s instructions^68^. Three million chondrocytes were fixed in 37% formaldehyde, pelleted, and resuspended in lysis buffer. The cells were sonicated and centrifuged to remove insoluble material. The supernatants were collected, and protein G magnetic beads were added and incubated for 1 h at 4 °C with anti-*HPIP* (Proteintech, 1:50, 12102-1-AP), anti-*LEF1* (Abcam, 1:50, ab137872), *H3K4ac* (Abcam, 1:50, ab176799), *H3K9ac* (Abcam, 1:50, ab10812), *H3K14ac* (Abcam, 1:50, ab52946), *H3K56ac* (Abcam, 1:25, ab71956). The chromatin was collected, purified, and de-crosslinked at 62 °C for 2 h, followed by incubation at 95 °C for 10 min. The DNA was isolated using the ChIP DNA Clean & Concentrator kit (Zymo Research Corp.) according to the manufacturer’s instructions. The precipitated DNA fragments were quantified through RT-PCR analysis. For re-ChIP, complexes were eluted from the primary immunoprecipitation by incubation with 10 mM DTT at 37 °C for 30 min and diluted 1:50 in re-ChIP buffer (150 mM NaCl, 1% Triton X-100, 2 mM EDTA, 20 mM Tris–HCl, pH 8.1) followed by re-immunoprecipitation with the second antibodies. RT-PCR was performed to detect relative occupancy.

### GST pull-down and coimmunoprecipitation assays

For the GST pull-down assay, GST-fusion proteins were expressed and purified according to the manufacturers’ instructions (Amersham Pharmacia and Qiagen). Cell lysates expressing target proteins were incubated with GST-fusion proteins bound to GST beads (Amersham Pharmacia), and the pull-down proteins were examined. For the coimmunoprecipitation assay, cells were harvested and lysed in lysis buffer on ice for 30 min. After centrifugation at 4 °C at 13,800 × *g* for 15 min, antibodies were added to the supernatant with rolling at 4 °C overnight. Protein G or A agarose (Santa Cruz) was then added to the samples, and the samples were rolled at 4 °C for 2 h. After the beads were washed three times with lysis buffer, the pellets were dissolved into 2 × SDS loading buffer after centrifugation and boiled at 100 °C for 10 min. Proteins were analyzed by immunoblotting with the indicated antibodies.

### Statistical analysis

Statistical analysis of the RNA-seq data was performed, and a two-tailed *t*-test with the Benjamini and Hochberg correction was used. All data were log transformed. GO terms with corrected *P*-values <0.05 were considered significantly enriched by differentially expressed genes. Evaluation of quantitative RT-PCR data was performed using one-way ANOVA with Tukey’s post hoc test. All analyses were performed using SPSS software 17.0 or GraphPad PRISM 6 (GraphPad). The data are expressed as the mean ± standard deviation, and *P* *<* 0.05 was considered statistically significant.

## Supplementary information

Supplementary Information

Reporting Summary

## Data Availability

All relevant data is available from the authors. The RNA-seq data and the complete ChIP-seq datasets in this study have been deposited and released at Gene Expression Omnibus (GEO) ([http://www.ncbi.nlm.nih.gov/geo/]) under accession ID GSE100312.
