# Supplementary material for: Hematopoietic PBX-interacting protein mediates cartilage degeneration during the pathogenesis of osteoarthritis
Source: Nat Commun. 2019 Jan 18;10:313. doi: 10.1038/s41467-018-08277-5 (PMC6338798; doi:10.1038/s41467-018-08277-5)
Supplement: Supplementary file 1 — Supplementary Information [file 41467_2018_8277_MOESM1_ESM.pdf]

SUPPLEMENTARY INFORMATION

Hematopoietic PBX-interacting protein mediates cartilage degeneration during the pathogenesis of osteoarthritis

Ji et al.

Supplementary Figure 1

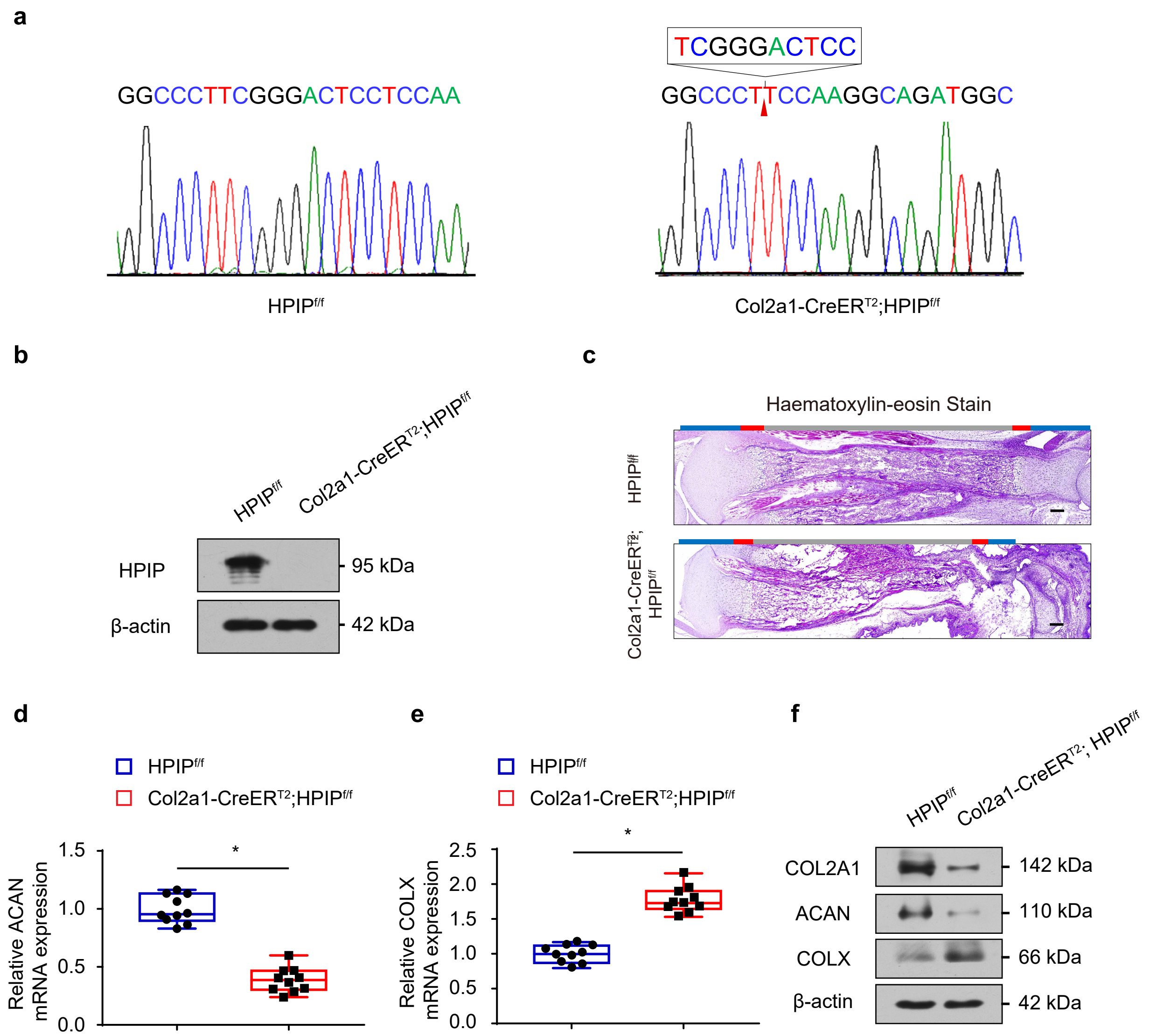

Supplementary Figure 1. Identification of HPIP deficiency. (**a**) DNA sequencing analysis of *HPIP*$^{f/f}$ and *Col2a1-CreER*^{T2}*;HPIP*$^{f/f}$ mice. (**b**) Immunoblots were incubated with the indicated antibodies. (**c**) Haematoxylin-eosin staining of whole tibias of *HPIP*$^{f/f}$ and *Col2a1-CreER*^{T2}*;HPIP*$^{f/f}$ littermate embryos (P1) are shown. Scale bar, 100 μm. (**d**) qRT-PCR measurement of ACAN of the proximal tibias of *HPIP*$^{f/f}$ (n = 10) and *Col2a1-CreER*^{T2}*;HPIP*$^{f/f}$ (n = 10) littermate embryos (P1). (**e**) qRT-PCR measurement of COLX of the proximal tibias of *HPIP*$^{f/f}$ (n = 10) and *Col2a1-CreER*^{T2}*;HPIP*$^{f/f}$ (n = 10) littermate embryos (P1). Center value represents the median of the HPIP scores. The bounds of box represent the upper quartile and the lower quartile. The whiskers represent the maximum and minimum score. Error bar represents the standard deviation (s.d.) and *P* value was generated by using one-way ANOVA with Tukey's post hoc test. *$P < 0.05$; **$P < 0.01$. (**f**) Immunoblot analysis with the indicated antibodies of the proximal tibias of *HPIP*$^{f/f}$ and *Col2a1-CreER*^{T2}*;HPIP*$^{f/f}$ littermate embryos (P1).

Supplementary Figure 2

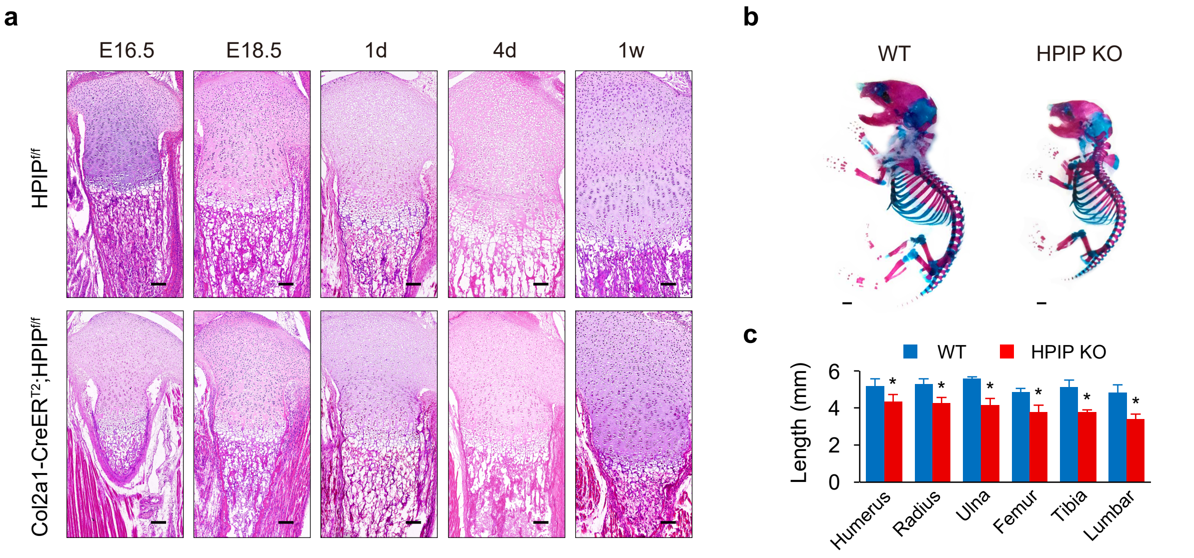

Supplementary Figure 2. Identification of the skeletal and cartilage abnormalities in mice. (**a**) Time course of Haematoxylin-eosin staining in proximal tibias of the two genotypes from E16.5 to 1 week postnatal. Scale bars, 100 µm. d, day; w, week. (**b**) Double staining with alizarin red and alcian blue of the whole skeleton. Scale bar, 2 mm. WT, wild type; KO, knockout. (**c**) The length of long bones and vertebra (first to fifth lumbar spines) of WT (n = 10) and HPIP KO (n = 10) littermate embryos (P1).Error bar represents the standard deviation (s.d.) and *P* value was generated by using one-way ANOVA with Tukey's post hoc test.

Supplementary Figure 3

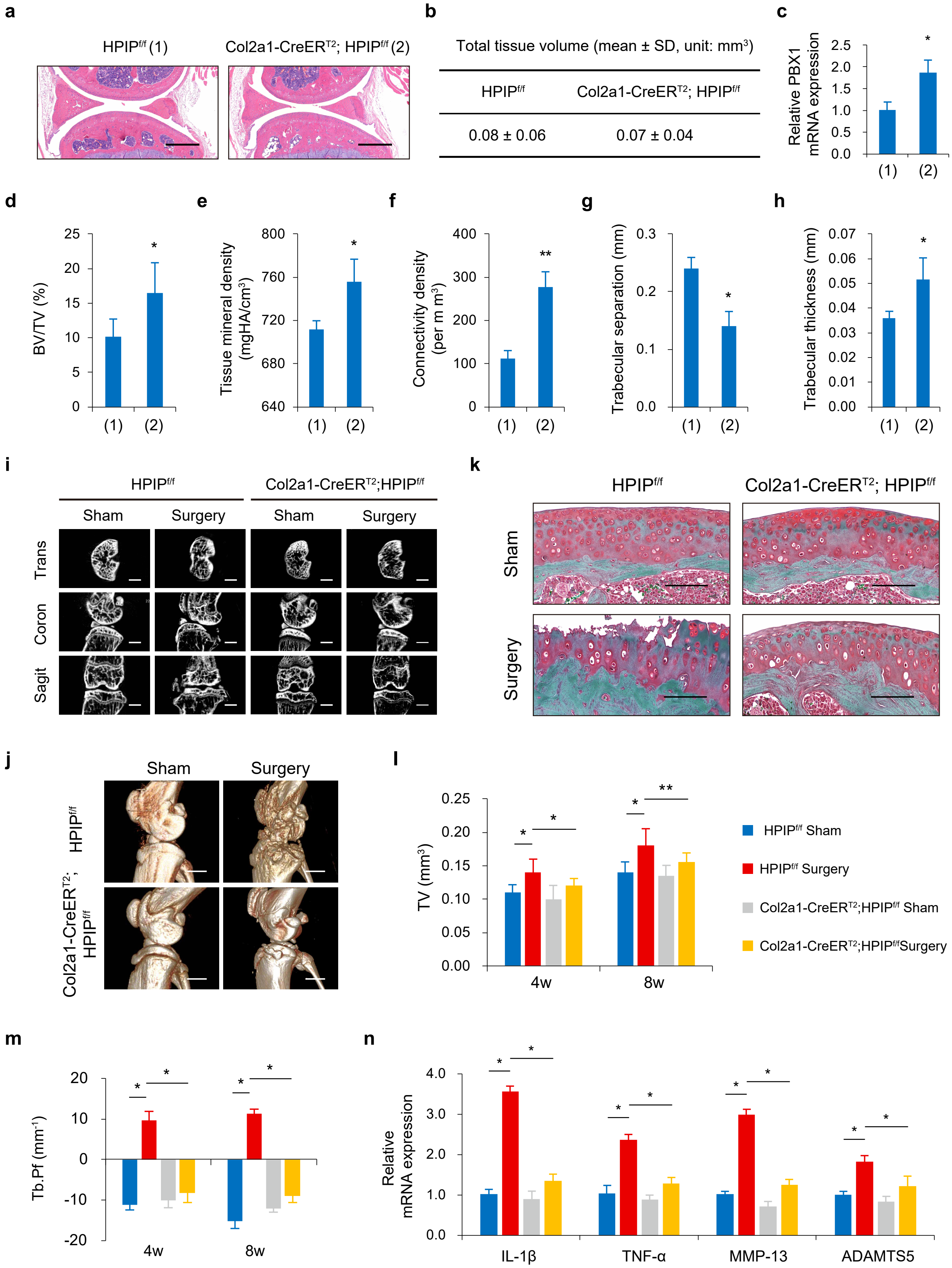

Supplementary Figure 3. *HPIP* ablation protects mice from the progression of OA. (**a**) Haematoxylin-eosin staining of the *HPIP*$^{f/f}$ and *Col2a1-CreER*^{T2}*;HPIP*$^{f/f}$ littermates at 8 weeks. (**b**) Quantitative analysis of total tissue volume in subchondral bone plate of the *HPIP*$^{f/f}$ (n = 10) and *Col2a1-CreER*^{T2}*;HPIP*$^{f/f}$ littermates (n = 10) at 8 weeks using μCT (n = 10). (**c**) qRT-PCR examination of *PBX1* in *HPIP*$^{f/f}$ (n = 10) and *Col2a1-CreER*^{T2}*;HPIP*$^{f/f}$ littermates (n = 10) at 8 weeks. (**d-h**) Quantitative analysis of bone volume fraction (BV/TV=bone volume/total volume)(d), tissue mineral density (e), connectivity density (f), trabecular separation (g) and trabecular thickness (h) in the *HPIP*$^{f/f}$ (n = 10) and *Col2a1-CreER*^{T2}*;HPIP*$^{f/f}$ littermates (n = 10) at 8 weeks using μCT. (**i**) Representative μCT images of the transverse (Trans), coronal (Coron) and sagittal (Sagit) views of the knee joints. Scale bar, 1 mm. (**j**) Three dimensional reconstruction analysis of μCT. Scale bar, 1 mm. (**k**) For mice at 8 weeks after ACLT surgery, the Safranin O/fast green staining was performed. Scale bar, 250 μm. (**l**) For mice at 8 weeks after ACLT surgery, quantitative analysis of total tissue volume (TV) in subchondral bone determined by μCT analysis (n = 10). (**m**) For mice at 8 weeks after ACLT surgery, quantitative analysis of trabecular pattern factor (Tb.Pf) in subchondral bone determined by μCT analysis (n = 10). (**n**) qRT-PCR examination of *IL-1β*, *TNF*-α, *MMP*-13 and *ADAMTS5* in *HPIP*$^{f/f}$ and *Col2a1-CreER*^{T2}*;HPIP*$^{f/f}$ littermate (8 weeks after ACLT). Error bar represents the standard deviation (s.d.) and *P* value was generated by using one-way ANOVA with Tukey's post hoc test. *$P < 0.05$; **$P < 0.01$.

Supplementary Figure 4

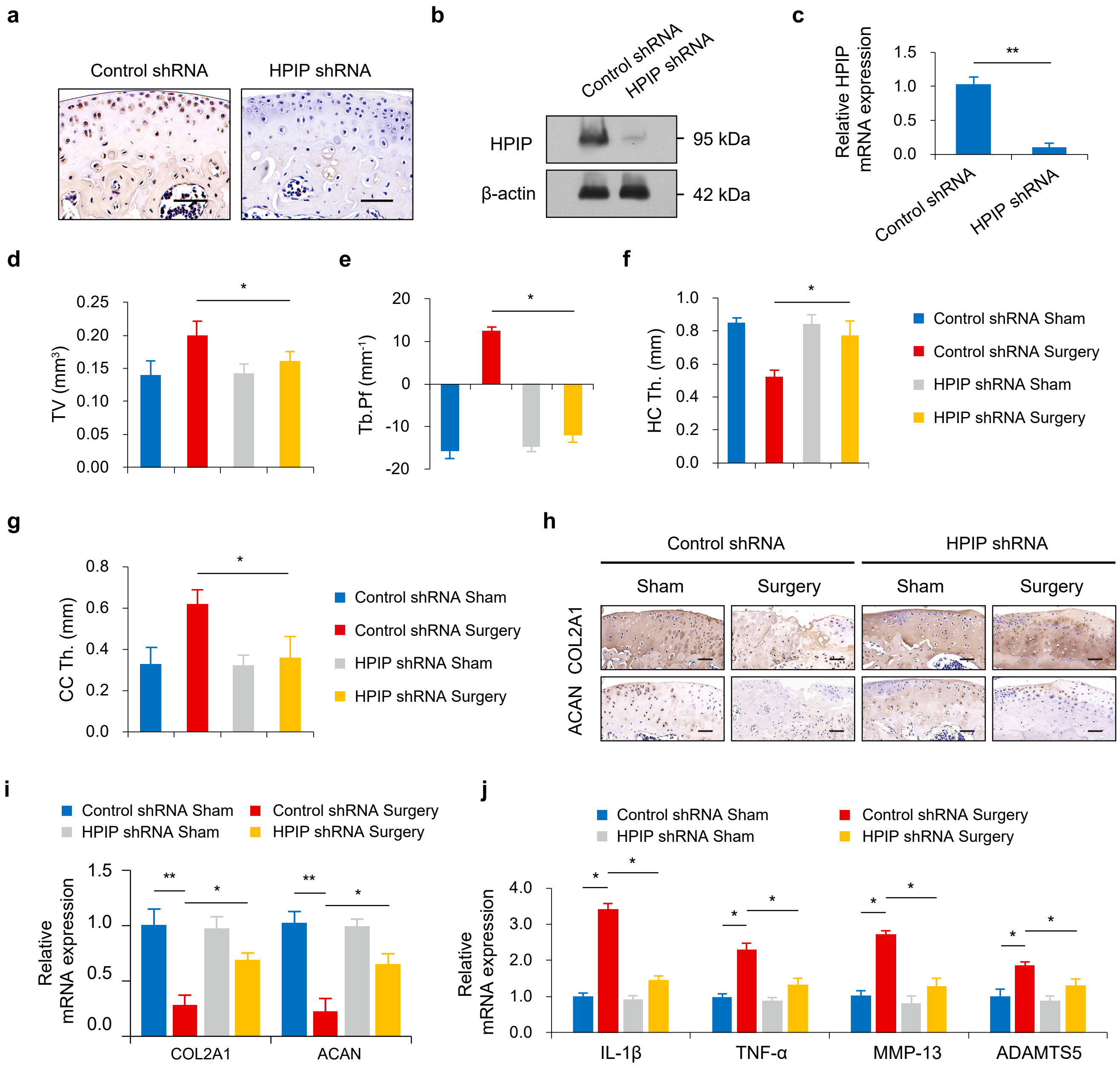

Supplementary Figure 4. Gene transfer with *HPIP*-specific shRNA for treating OA. (**a**) Immunohistochemistry assay with the indicated antibodies in C57/BL6J mice (8 weeks old) (n = 10) injected intra-articularly with AAV carrying *HPIP*-specific shRNA. Scale bar, 50 μm. (**b,c**) Immunoblot analysis (b) and qRT-PCR examination (c) of *HPIP* in the proximal tibias of C57/BL6J mice (8 weeks old) (n = 10) injected intra-articularly with AAV carrying *HPIP*-specific shRNA. (**d-g**) For mice at 8 weeks after ACLT surgery, quantitative analysis of total tissue volume (TV) (d), trabecular pattern factor (Tb.Pf) (e), thickness of hyaline cartilage (HC) (HC Th.) (f) and thickness of calcified cartilage (CC) (CC Th.) (g) in subchondral bone (n = 10). (**h,i**) Immunohistochemistry assay with the indicated antibodies (h) and qRT-PCR examination (i) in mice 8 weeks after ACLT(n = 10). Scale bar, 100 μm. (**j**) qRT-PCR examination of *IL-1β*, *TNF-α*, *MMP-13* and *ADAMTS5* in mice 8 weeks after ACLT (n = 10). Error bar represents the standard deviation (s.d.) and P value was generated by using one-way ANOVA with Tukey's post hoc test. *$P < 0.05$; **$P < 0.01$.

Supplementary Figure 5

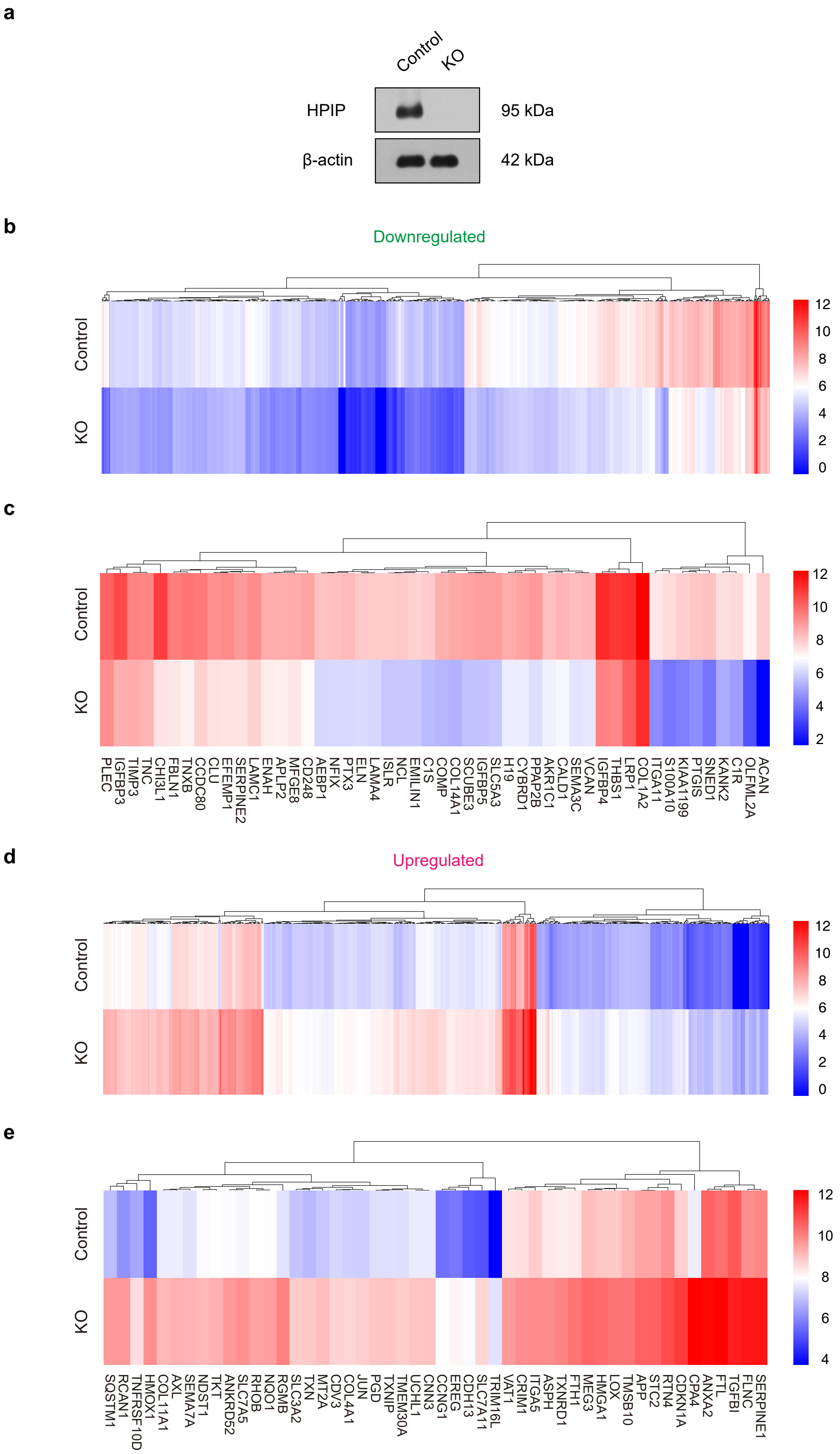

Supplementary Figure 5. Hierarchical clustering of RNA-seq analysis between the control and *HPIP* knockout (KO) chondrocytes. (**a**) Immunoblot analysis of *HPIP* in the control and *HPIP* KO chondrocytes. (**b,c**) The hierarchical clustering of RNAseq analysis showing the genes downregulated between the control and *HPIP* KO groups. The top 50 genes with altered expression are shown. (**d,e**) The hierarchical clustering of RNA-seq analysis showing the genes upregulated between the control and *HPIP* KO groups. The top 50 genes with altered expression are shown.

Supplementary Figure 6

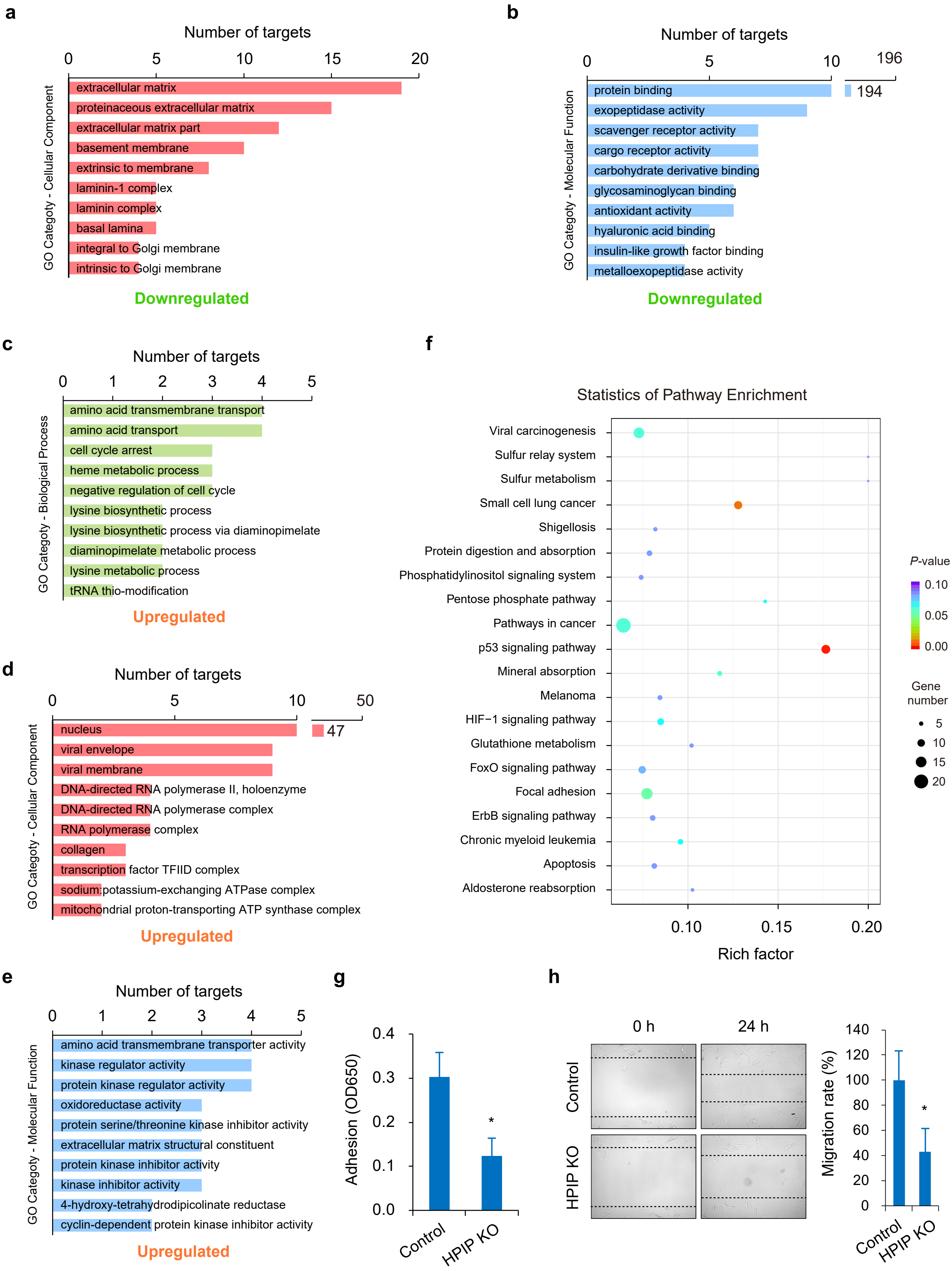

Supplementary Figure 6. Gene Ontology (GO) enrichment and KEGG pathway analysis of genes regulated by *HPIP*. (**a,b**) The GO functional clustering of genes downregulated for cellular component and molecular function in *HPIP* deficiency (top 10 most significantly affected categories are shown). (**c-e**) GO functional clustering of genes upregulated for biological process, cellular component and molecular function in *HPIP* deficiency (top 10 most significantly affected categories are shown). (**f**) The KEGG pathway analysis of upregulated targets in *HPIP* deficiency. (**g**) Cell adhesion assay in the control and *HPIP* KO chondrocytes. (**h**) Migration assays were conducted in the control and *HPIP* KO chondrocytes. Cell migration was measured 24h after the cell layers were scratched. Error bar represents the standard deviation (s.d.) and *P* value was generated by using one-way ANOVA with Tukey's post hoc test. $*P < 0.05$; $**P < 0.01$.

Supplementary Figure 7

a

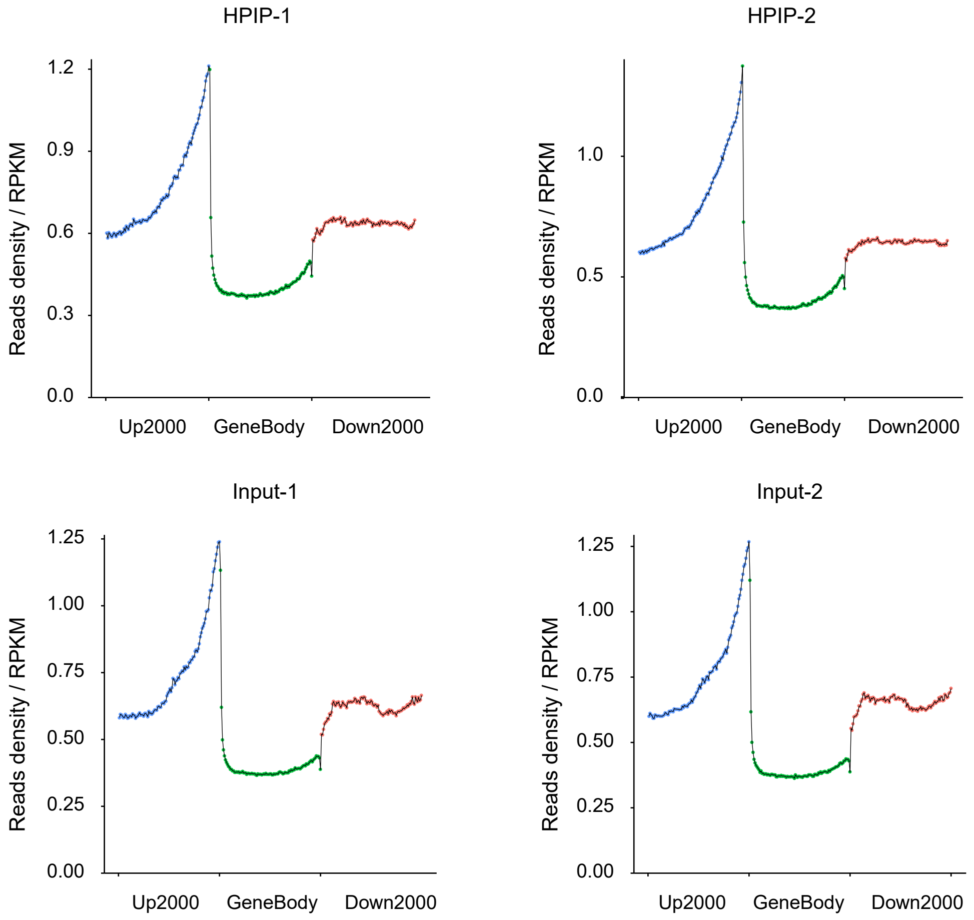

b

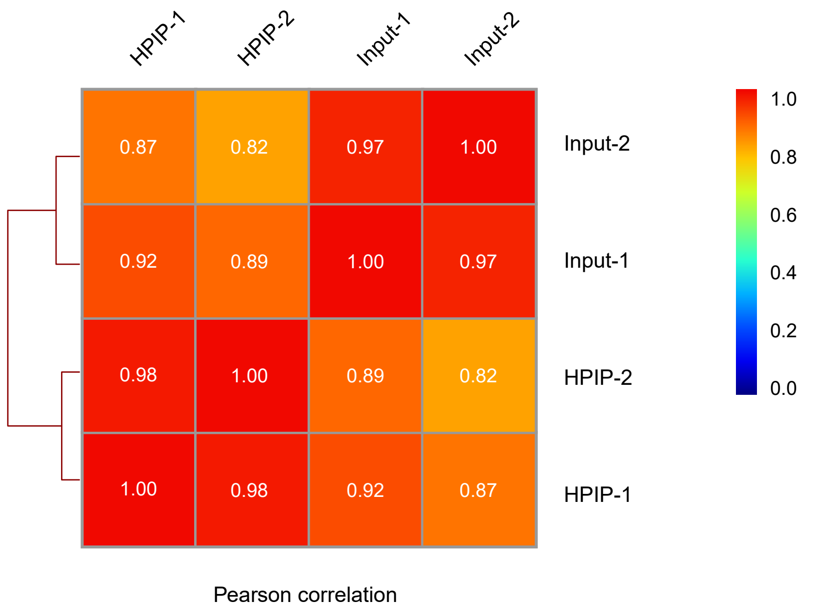

Supplementary Figure 7. Quality evaluation of *HPIP* ChIP-seq assay. (**a**) Relative gene position distribution of reads segments. Each gene and the gene’s upstream and downstream 2000 bp were divided into 100 bins separately. The number of reads in each bin was calculated to be the percentage of total reads in these regions, which were represented as the reads density of each bin. RPKM, Reads Per Kilobase of transcript per Million mapped reads. (**b**) Pearson correlation analysis of the different ChIP-seq groups.

Supplementary Figure 8

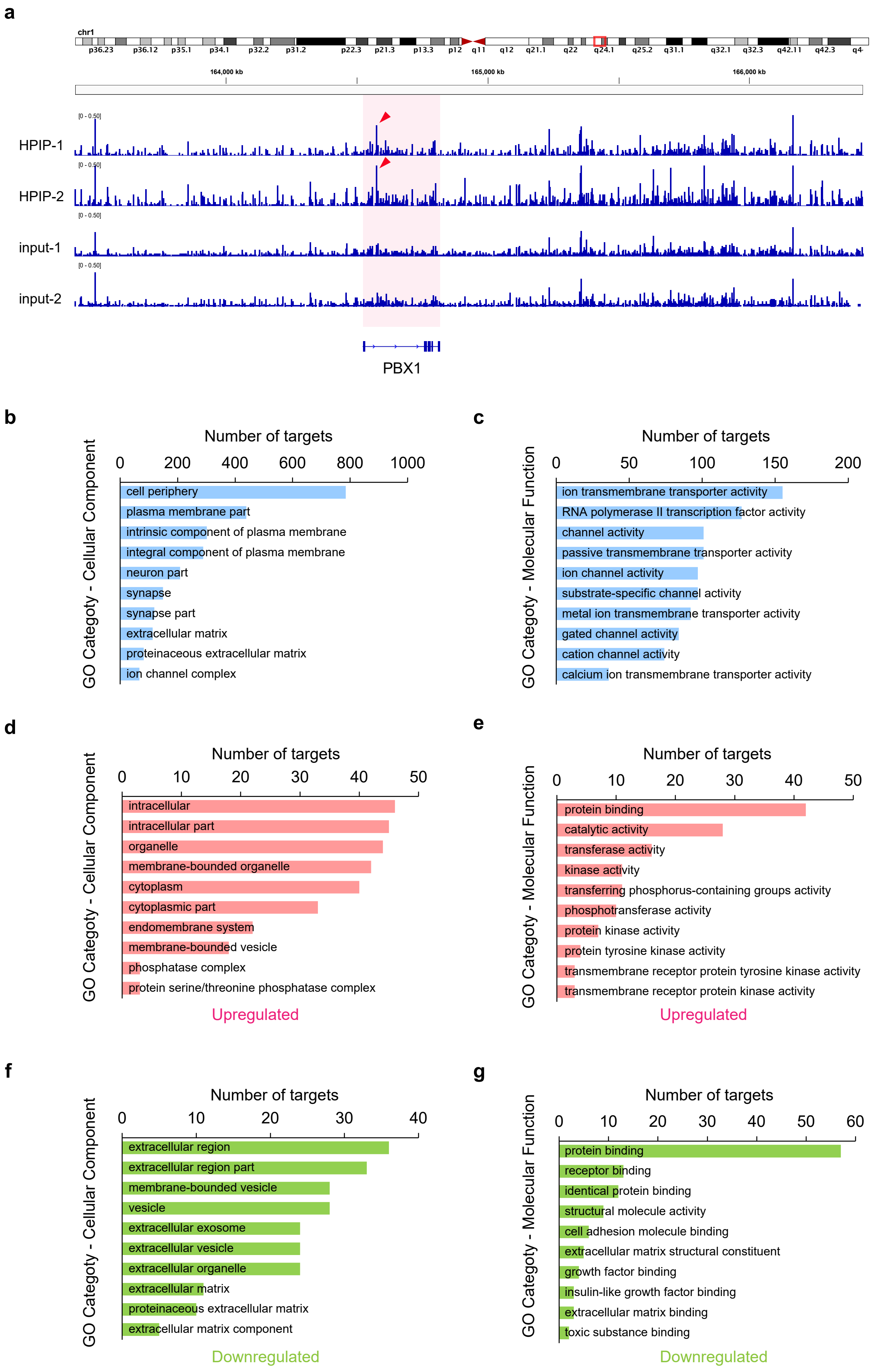

Supplementary Figure 8. Analysis of targets associated with *HPIP* ChIP-seq peaks. (**a**) ChIP-seq data from *PBX1*. Genome Browser display of the *PBX1* locus on chr1 from the human assembly. (**b,c**) The GO functional clustering of targets associated with *HPIP* ChIP-seq peaks for cellular component and molecular function (top 10 most significantly affected categories are shown). (**d,e**) The GO functional clustering of targets upregulated for identification of cellular component and molecular function directly regulated by *HPIP* (top 10 most significantly affected categories are shown). (**f,g**) The GO functional clustering of targets downregulated for identification of cellular component and molecular function directly regulated by *HPIP* (top 10 most significantly affected categories are shown).

Supplementary Figure 9

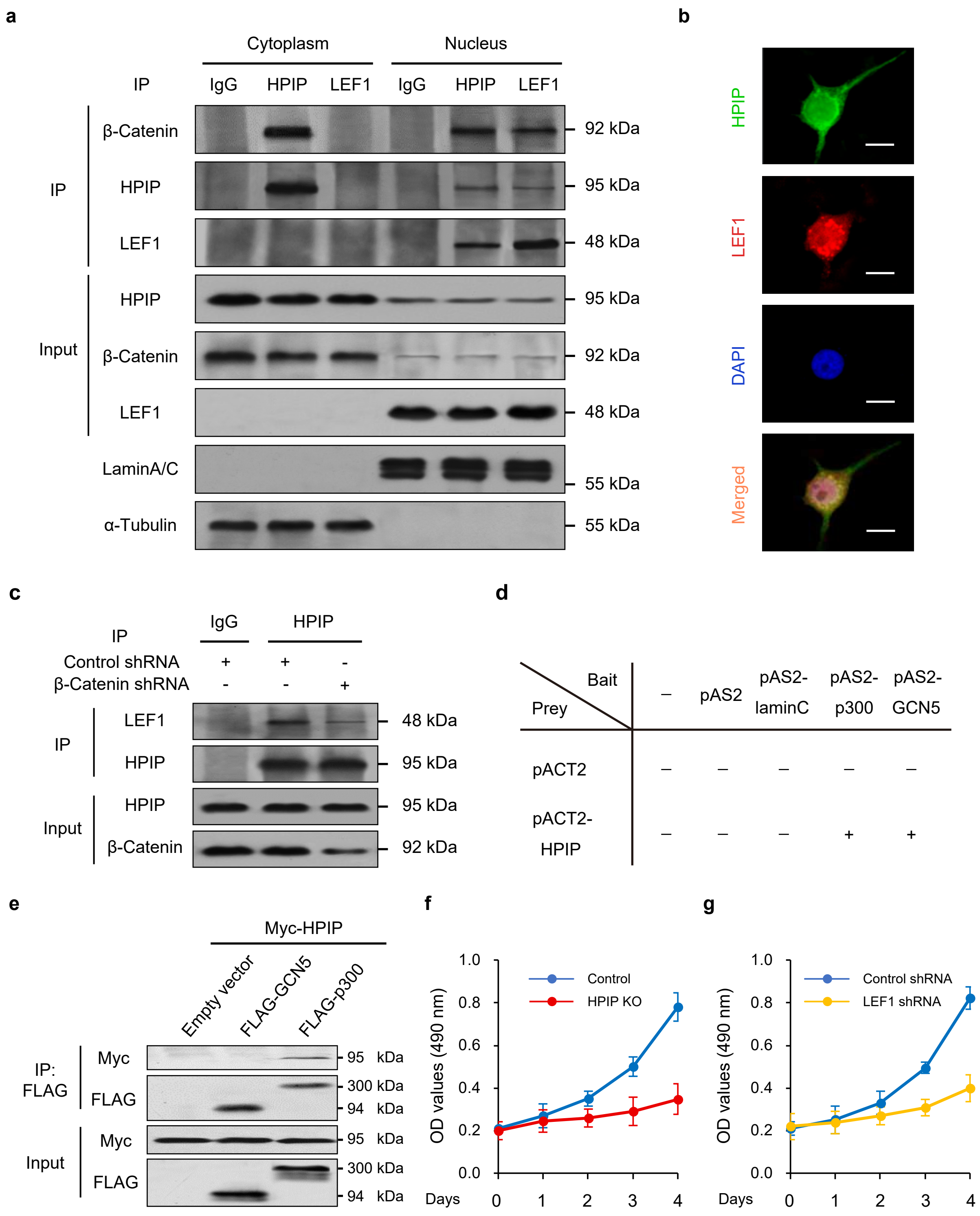

Supplementary Figure 9. *HPIP* physically interacts with *LEF1*. (**a**) Chondrocytes were fractionated into cytoplasmic and nuclear fractions, and IP with the indicated antibodies or control serum (IgG). Precipitates were analysed by immunoblot using the indicated antibodies. *Lamin A/C* and α-*tubulin* were used as the nuclear and cytoplasmic marker, respectively. (**b**) Immunofluorescence analysis of chondrocytes treated with the indicated antibodies. Cells were stained with anti-*HPIP* (green) and anti-*LEF1* (red). The nuclei were stained with DAPI (blue). If green and red colours overlap, yellow colour appears. Scale bars, 100 μm. (**c**) Chondrocytes stably infected with *β-catenin* shRNA or control shRNA and IP with anti-*HPIP* or normal IgG. The resulting precipitates were analysed by immunoblotting with the indicated antibodies. IP, immunoprecipitation. (**d**) Yeast CG1945 cells were transformed with the indicated plasmids (bait and prey for the two-hybrid assay) and grown on selective media. Positive interaction is indicative of colonies that grow on selective media and have β-galactosidase activity. (**e**) Chondrocytes infected with the indicated plasmids were IP with anti-*FLAG*. (**f,g**) Chondrocytes in each indicated group were cultured in regular medium. At the specified times, cells were determined with the CCK-8 assay. Error bar represents the standard deviation (s.d.).

Supplementary Figure 10

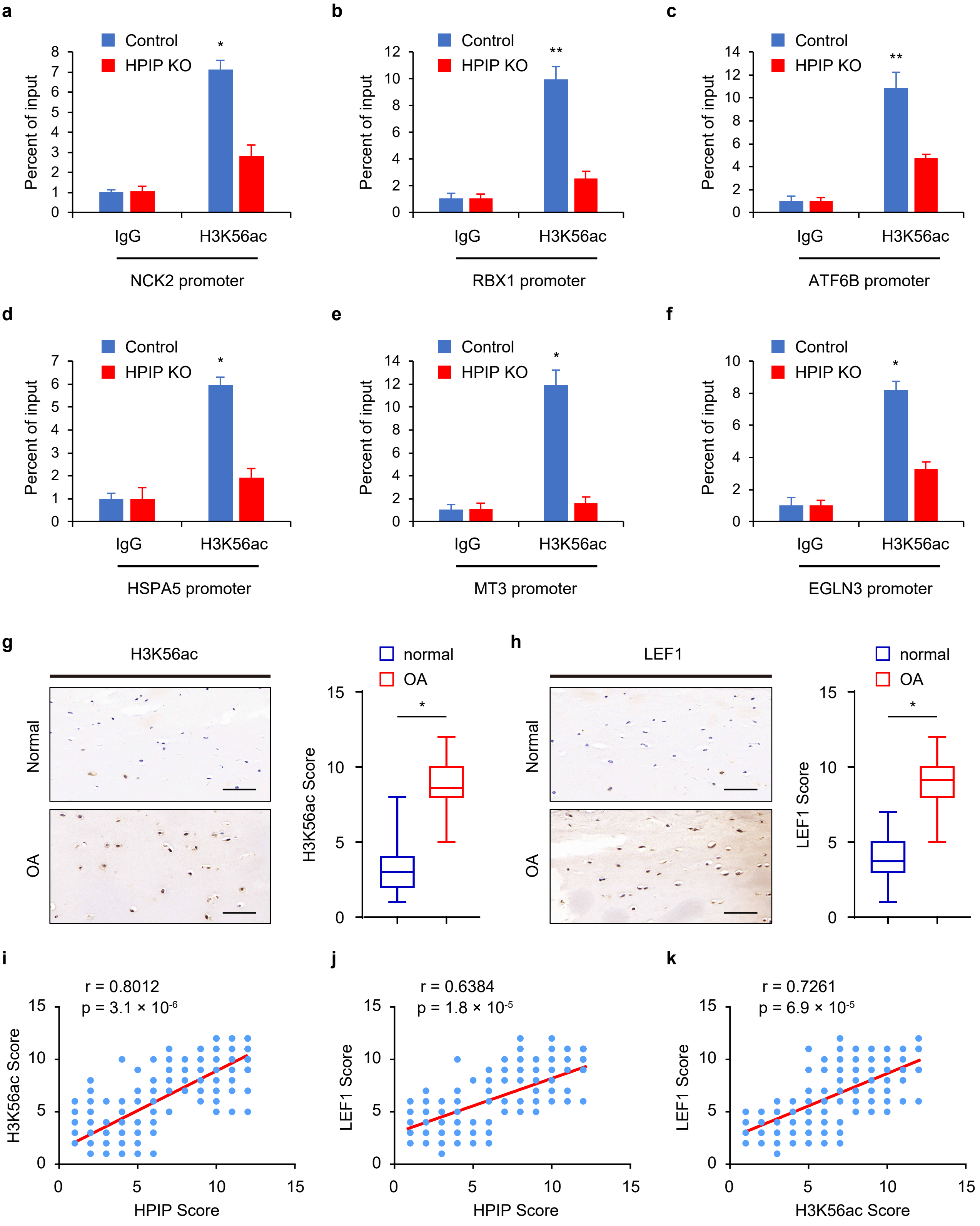

Supplementary Figure 10. *HPIP* physically interacts with *LEF1* and acetylates *H3K56ac* at the promoters of *Wnt* target genes in OA and the expression of *H3K56ac* or *LEF1* in OA patients. (**a-f**) ChIP analysis of *H3K56ac* occupancy on the promoters of DNA replication and stresses response genes which were derived from RNA-seq data in *HPIP* knockout (KO) chondrocytes. (**g**) Representative immunohistochemistry images of *H3K56ac* expression in normal tissues and OA cartilage tissues. Scale bars: 500 μm. *H3K56ac* expression scores in normal tissues and OA cartilage tissues were compared with the Mann–Whitney U-test (Right). (**h**) Representative immunohistochemistry images of *LEF1* expression in normal tissues and OA cartilage tissues. Scale bars: 50 μm. *LEF1* expression scores in normal tissues and OA cartilage tissues were compared with the Mann–Whitney U-test (Right). Center value represents the median of the *HPIP* scores. The bounds of box represent the upper quartile and the lower quartile. The whiskers represent the maximum and minimum score. Error bar represents the standard deviation (s.d.). *$P < 0.05$; **$P < 0.01$. (**i**) The relationship between *HPIP* and *H3K56ac* expression score was assessed by Spearman's rank correlation analysis in the cartilage samples. (**j**) The relationship between *HPIP* and *LEF1* expression score was assessed by Spearman's rank correlation analysis in the cartilage samples. (**k**) The relationship between *LEF1* and *H3K56ac* expression score was assessed by Spearman's rank correlation analysis in the cartilage samples. The symbols represent individual samples.

Supplementary Figure 11

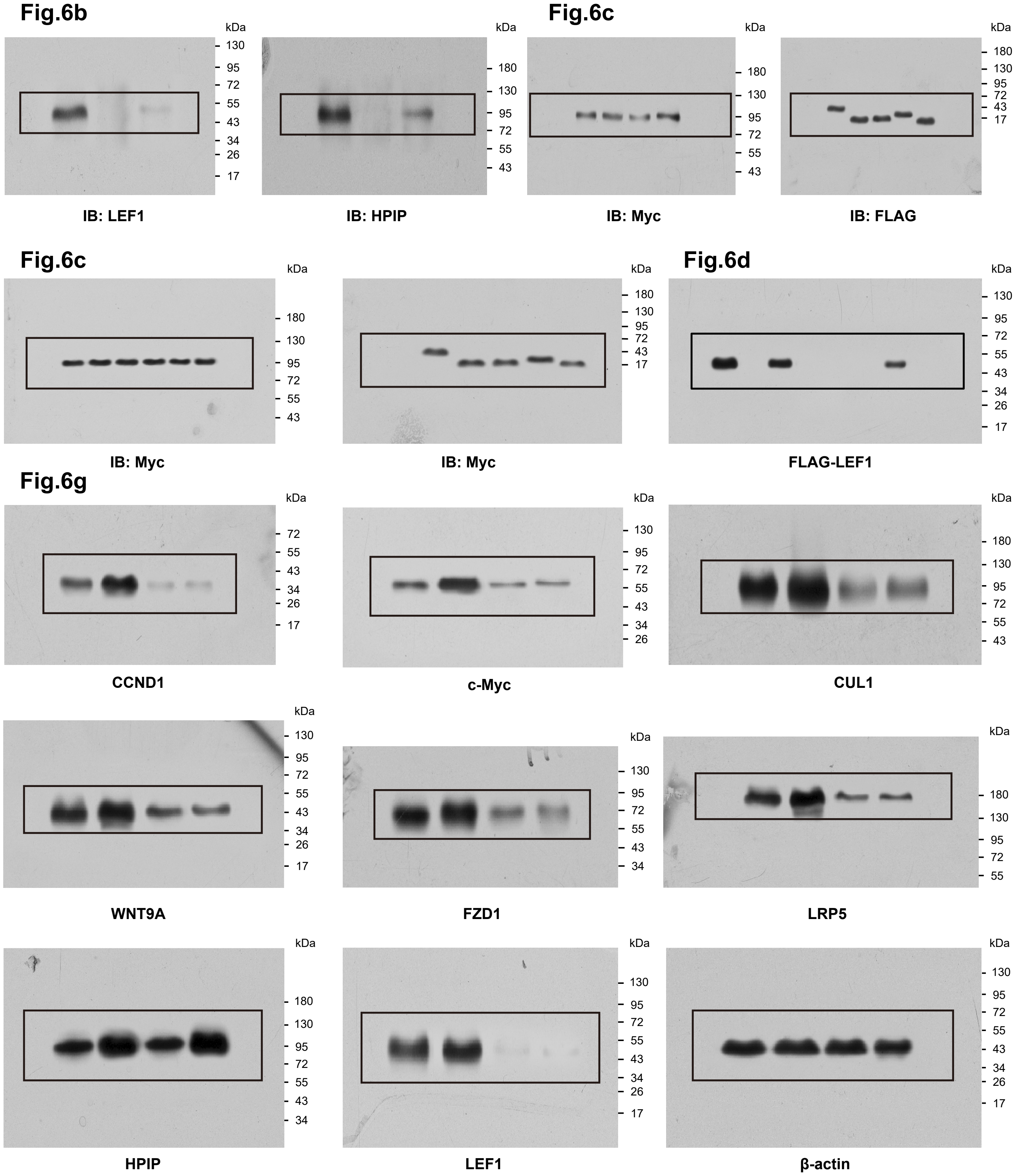

Supplementary Figure 10. Uncropped images of the most important blots.

Supplementary Table 1 Clinical and demographic characteristics of the study population*

**Clinical data**	
Age, years	63.4 ± 5.6
Male/ female (n)	52/66
Disease duration, years	10.3 ± 5.4
Swollen joints (n)	1.2 ± 0.8
Tender joints (n)	1.1 ± 0.7
HSS score [a]	46.7 ± 6.6
CRP, mg/dl	0.5 ± 0.3
ESR, mm/h	13.8 ± 7.2

* Data are shown as the mean ± SD or absolute numbers.

[a] The Hospital for Special Surgery Knee Score (HSS) was used. Scores can change from 0 to 100, with lower scores suggesting greater disease activity.

CRP, C-reactive protein; ESR, erythrocyte sedimentation rate.

Supplementary Table 2 Cartilage thickness in different group and time-points

Time	Hyaline cartilage				Calcified cartilage			
(weeks)	$HPIP^{f/f}$		Col2a1-$CreER^{T2}$; $HPIP^{f/f}$		$HPIP^{f/f}$		Col2a1-$CreER^{T2}$; $HPIP^{f/f}$	
	sham	surgery	sham	surgery	sham	surgery	sham	surgery
4	0.83±0.03	0.74±0.06*	0.81±0.02	0.80±0.02#	0.33±0.04	0.42±0.03*	0.32±0.05	0.34±0.07#
8	0.84±0.09	0.59±0.02*	0.83±0.06	0.81±0.04#	0.34±0.11	0.59±0.14*	0.33±0.09	0.36±0.12#

The data are expressed as the mean ± standard deviation, unit: mm. $P < 0.05$ was considered statistically significant. The comparison between *$HPIP^{f/f}$* sham group and *$HPIP^{f/f}$* surgery group was indicated by '*' and the comparison between *Col2a1-$CreER^{T2}$; $HPIP^{f/f}$* surgery group and *$HPIP^{f/f}$* surgery group group was indicated by '＃'.

Supplementary Table 3 List of the top 50 downregulated genes following HPIP deficiency

Gene symbol	Description	Gene ID	Fold difference	P-value
CFH	complement factor H	ENSG00000000971	-1.7679	3.14E-289
SEMA3F	sema domain, immunoglobulin domain (Ig), short basic domain, secreted, (semaphorin) 3F	ENSG00000001617	-1.7464	2.62E-287
CYP26B1	cytochrome P450, family 26, subfamily B, polypeptide 1	ENSG00000003137	-1.2551	1.23E-286
RECQL	RecQ protein-like (DNA helicase Q1-like)	ENSG00000004700	-1.0279	5.34E-285
HSPB6	heat shock protein, alpha-crystallin-related, B6	ENSG00000004776	-1.4822	4.38E-283
PDK4	pyruvate dehydrogenase kinase, isozyme 4	ENSG00000004799	-2.2984	5.26E-283
CDC27	cell division cycle 27	ENSG00000004897	-1.2965	9.84E-281
COPZ2	coatomer protein complex, subunit zeta 2	ENSG00000005243	-1.3365	4.16E-269
CRLF1	cytokine receptor-like factor 1	ENSG00000006016	-2.2403	2.03E-233
CACNA1G	calcium channel, voltage-dependent, T type, alpha 1G subunit	ENSG00000006283	-4.647	1.63E-225
ALDH3B1	aldehyde dehydrogenase 3 family, member B1	ENSG00000006534	-1.9275	3.51E-223
PHTF2	putative homeodomain transcription factor 2	ENSG00000006576	-1.9643	5.02E-217
NFIX	nuclear factor I/X (CCAAT-binding transcription factor)	ENSG00000008441	-2.3716	8.93E-206
SEC62	SEC62 homolog	ENSG00000008952	-2.2385	2.31E-198
BAZ1B	bromodomain adjacent to zinc finger domain, 1B	ENSG00000009954	-1.9577	2.02E-177
CD9	CD9 molecule	ENSG00000010278	-1.9904	5.73E-161
SYT7	synaptotagmin VII	ENSG00000011347	-5.2579	3.63E-147
RABGAP1	RAB GTPase activating protein 1	ENSG00000011454	-1.4273	6.38E-146
DCN	decorin	ENSG00000011465	-1.5458	4.40E-139
SEMA3B	sema domain, immunoglobulin domain (Ig), short basic domain, secreted, (semaphorin) 3B	ENSG00000012171	-2.7128	5.66E-127
MBTPS2	membrane-bound transcription factor peptidase, site 2	ENSG00000012174	-2.1952	5.17E-122
CALCOCO1	calcium binding and coiled-coil domain 1	ENSG00000012822	-1.3167	3.25E-117
MAN2B2	mannosidase, alpha, class 2B, member 2	ENSG00000013288	-1.3619	1.87E-113
NUB1	negative regulator of ubiquitin-like proteins 1	ENSG00000013374	-1.331	2.44E-113
UFL1	UFM1-specific ligase 1	ENSG00000014123	-1.6029	9.98E-113

Gene symbol	Description	Gene ID	Fold difference	P-value
CD248	CD248 molecule, endosialin	ENSG00000174807	-1.4221	1.54E-112
PLEC	plectin	ENSG00000178209	-1.5548	1.73E-111
PTX3	pentraxin 3, long	ENSG00000163661	-1.1909	2.91E-101
VCAN	versican	ENSG00000038427	-1.6406	6.55E-92
S100A10	S100 calcium binding protein A10	ENSG00000197747	-1.8528	6.26E-91
NFIX	nuclear factor I/X (CCAAT-binding transcription factor)	ENSG00000008441	-1.0056	1.08E-88
C1S	complement component 1, s subcomponent	ENSG00000182326	-1.649	1.07E-86
AEBP1	AE binding protein 1	ENSG00000106624	-2.1292	1.37E-85
SEMA3C	sema domain, immunoglobulin domain (Ig), short basic domain, secreted, (semaphorin) 3C	ENSG00000075223	-1.3572	6.48E-81
NCL	nucleolin	ENSG00000115053	-1.819	2.38E-80
CALD1	caldesmon 1	ENSG00000122786	-1.8166	9.29E-80
C1R	complement component 1, r subcomponent	ENSG00000159403	-1.394	7.89E-78
ISLR	immunoglobulin superfamily containing leucine-rich repeat	ENSG00000129009	-1.5525	4.92E-77
TNC	tenascin C	ENSG00000041982	-1.2619	2.73E-76
TIMP3	TIMP metallopeptidase inhibitor 3	ENSG00000100234	-2.2404	1.14E-74
LAMA4	laminin, alpha 4	ENSG00000112769	-1.3402	1.48E-66
EMILIN1	elastin microfibril interfacer 1	ENSG00000138080	-1.7607	5.63E-66
APLP2	amyloid beta (A4) precursor-like protein 2	ENSG00000084234	-1.1022	1.74E-65
ITGA11	integrin, alpha 11	ENSG00000137809	-1.0504	4.34E-64
ENAH	enabled homolog	ENSG00000154380	-2.5629	7.13E-64
ELN	elastin	ENSG00000049540	-3.3944	2.04E-63
KANK2	KN motif and ankyrin repeat domains 2	ENSG00000197256	-2.8391	1.45E-62
OLFML2A	olfactomedin-like 2A	ENSG00000185585	-1.5776	5.36E-62
AKR1C1	aldo-keto reductase family 1, member C1	ENSG00000187134	-2.0123	5.82E-62
MFGE8	milk fat globule-EGF factor 8 protein	ENSG00000140545	-1.4124	8.77E-62

Supplementary Table 4 List of the top 50 upregulated genes following HPIP deficiency

Gene symbol	Description	Gene ID	Fold difference	P-value
FTL	ferritin, light polypeptide	ENSG00000087086	1.9199	4.32E-276
SERPINE1	serpin peptidase inhibitor, clade E (nexin, plasminogen activator inhibitor type 1), member 1	ENSG00000106366	1.9438	5.64E-269
CDKN1A	cyclin-dependent kinase inhibitor 1A	ENSG00000124762	2.5774	5.12E-268
CPA4	carboxypeptidase A4	ENSG00000128510	4.7089	6.18E-262
FLNC	filamin C, gamma	ENSG00000128591	2.0948	8.12E-260
ANXA2	annexin A2	ENSG00000182718	1.6334	1.28E-256
HMOX1	heme oxygenase (decycling) 1	ENSG00000100292	4.6075	7.01E-255
RCAN1	regulator of calcineurin 1	ENSG00000159200	3.6239	6.16E-200
RGMB	RGM domain family, member B	ENSG00000174136	2.6232	1.72E-196
TGFBI	transforming growth factor, beta-induced, 68kDa	ENSG00000120708	1.0191	1.38E-175
FTH1	ferritin, heavy polypeptide 1	ENSG00000167996	2.0097	1.38E-168
SQSTM1	sequestosome 1	ENSG00000161011	2.8673	1.26E-161
HMGA1	high mobility group AT-hook 1	ENSG00000137309	1.5867	1.94E-139
RTN4	reticulon 4	ENSG00000115310	1.2234	7.65E-135
MEG3	maternally expressed 3 (non-protein coding)	ENSG00000214548	1.4774	1.94E-128
TXNRD1	thioredoxin reductase 1	ENSG00000198431	1.8224	5.26E-125
SLC7A5	solute carrier family 7 (amino acid transporter light chain, L system), member 5	ENSG00000103257	2.036	2.90E-122
LOX	lysyl oxidase	ENSG00000113083	1.4784	5.51E-112
ANKRD52	ankyrin repeat domain 52	ENSG00000139645	1.8652	2.88E-101
SLC7A11	solute carrier family 7 (anionic amino acid transporter light chain, xc- system), member 11	ENSG00000151012	3.6833	5.01E-100
STC2	stanniocalcin 2	ENSG00000113739	1.1514	3.81E-95
APP	amyloid beta (A4) precursor protein	ENSG00000142192	1.1546	1.34E-94
ASPH	aspartate beta-hydroxylase	ENSG00000198363	1.6022	2.23E-94
NQO1	NAD(P)H dehydrogenase, quinone 1	ENSG00000181019	1.7066	8.64E-84
CRIM1	cysteine rich transmembrane BMP regulator 1	ENSG00000150938	1.3872	6.10E-75

Gene symbol	Description	Gene ID	Fold difference	P-value
MT2A	metallothionein 2A	ENSG00000125148	2.0474	1.60E-72
RHOB	ras homolog family member B	ENSG00000143878	1.5927	2.45E-72
TMSB10	thymosin beta 10	ENSG00000034510	1.0914	1.54E-69
SEMA7A	semaphorin 7A, GPI membrane anchor (John Milton Hagen blood group)	ENSG00000138623	1.7973	1.83E-69
TXN	thioredoxin	ENSG00000136810	2.1563	3.72E-69
AXL	AXL receptor tyrosine kinase	ENSG00000167601	1.6287	4.31E-62
ITGA5	integrin, alpha 5 (fibronectin receptor, alpha polypeptide)	ENSG00000161638	1.1579	4.73E-60
VAT1	vesicle amine transport protein 1 homolog	ENSG00000108828	1.2626	4.10E-56
SLC3A2	solute carrier family 3 (activators of dibasic and neutral amino acid transport), member 2	ENSG00000168003	1.8926	1.25E-55
TNFRSF10D	tumor necrosis factor receptor superfamily, member 10d, decoy with truncated death domain	ENSG00000173530	2.1263	2.08E-53
COL11A1	collagen, type XI, alpha 1	ENSG00000060718	1.5098	9.18E-52
EREG	epiregulin	ENSG00000124882	2.5272	2.87E-49
TRIM16L	tripartite motif containing 16-like	ENSG00000108448	4.0446	6.47E-48
JUN	jun proto-oncogene	ENSG00000177606	1.6957	1.16E-47
TXNIP	thioredoxin interacting protein	ENSG00000117289	1.5537	9.22E-47
PGD	phosphogluconate dehydrogenase	ENSG00000142657	1.5635	1.18E-46
TKT	transketolase	ENSG00000163931	1.3173	4.66E-46
CDH13	cadherin 13, H-cadherin	ENSG00000140945	2.7758	1.33E-45
NDST1	N-deacetylase/N-sulfotransferase (heparan glucosaminyl) 1	ENSG00000070614	1.3224	3.29E-43
CCNG1	cyclin G1	ENSG00000113328	2.4584	2.37E-42
CNN3	calponin 3, acidic	ENSG00000117519	1.3939	4.87E-41
UCHL1	ubiquitin carboxyl-terminal esterase L1 (ubiquitin thiolesterase)	ENSG00000154277	1.3658	3.98E-39
TMEM30A	transmembrane protein 30A	ENSG00000112697	1.4398	1.26E-38
COL4A1	collagen, type IV, alpha 1	ENSG00000187498	1.5387	1.46E-38
CDV3	CDV3 homolog	ENSG00000091527	1.4783	1.14E-37

Supplementary Table 5 List of the top 50 downregulated GO terms following HPIP deficiency

GO accession	Description	Term type	P-value
GO:0005515	protein binding	molecular_function	1.35E-06
GO:0022610	biological adhesion	biological_process	1.95E-06
GO:0007155	cell adhesion	biological_process	2.46E-06
GO:0040008	regulation of growth	biological_process	0.00011236
GO:0005044	scavenger receptor activity	molecular_function	0.00030292
GO:0038024	cargo receptor activity	molecular_function	0.00030292
GO:0030173	integral to Golgi membrane	cellular_component	0.00032502
GO:0031228	intrinsic to Golgi membrane	cellular_component	0.00032502
GO:0031012	extracellular matrix	cellular_component	0.00035501
GO:0005539	glycosaminoglycan binding	molecular_function	0.00039007
GO:0044420	extracellular matrix part	cellular_component	0.00047063
GO:0005578	proteinaceous extracellular matrix	cellular_component	0.00054664
GO:0097367	carbohydrate derivative binding	molecular_function	0.00077704
GO:0048870	cell motility	biological_process	0.00078378
GO:0051674	localization of cell	biological_process	0.00078378
GO:0005540	hyaluronic acid binding	molecular_function	0.00082615
GO:0001558	regulation of cell growth	biological_process	0.00082888
GO:0005520	insulin-like growth factor binding	molecular_function	0.00082888
GO:0005606	laminin-1 complex	cellular_component	0.00084951
GO:0016477	cell migration	biological_process	0.00084951
GO:0030155	regulation of cell adhesion	biological_process	0.00084951
GO:0030334	regulation of cell migration	biological_process	0.00084951
GO:0043256	laminin complex	cellular_component	0.00084951
GO:0045995	regulation of embryonic development	biological_process	0.00084951
GO:0051270	regulation of cellular component movement	biological_process	0.00084951

GO accession	Description	DEG-item	P-value
GO:2000145	regulation of cell motility	biological_process	0.00084951
GO:0005604	basement membrane	cellular_component	0.00087445
GO:0006979	response to oxidative stress	biological_process	0.0008987
GO:0048193	Golgi vesicle transport	biological_process	0.00098075
GO:0008235	metalloexopeptidase activity	molecular_function	0.00098968
GO:0040012	regulation of locomotion	biological_process	0.0010815
GO:0005605	basal lamina	cellular_component	0.0011107
GO:0016209	antioxidant activity	molecular_function	0.0011232
GO:0008238	exopeptidase activity	molecular_function	0.0011869
GO:2000026	regulation of multicellular organismal development	biological_process	0.0012472
GO:0006891	intra-Golgi vesicle-mediated transport	biological_process	0.0014178
GO:0019838	growth factor binding	molecular_function	0.0014695
GO:0016049	cell growth	biological_process	0.0015132
GO:0004601	peroxidase activity	molecular_function	0.0019724
GO:0016684	oxidoreductase activity, acting on peroxide as acceptor	molecular_function	0.0019724
GO:0050793	regulation of developmental process	biological_process	0.0029639
GO:0001871	pattern binding	molecular_function	0.0034448
GO:0030247	polysaccharide binding	molecular_function	0.0034448
GO:0009790	embryo development	biological_process	0.0038836
GO:0005507	copper ion binding	molecular_function	0.0044615
GO:0044765	single-organism transport	biological_process	0.0046967
GO:0004602	glutathione peroxidase activity	molecular_function	0.0049129
GO:0019898	extrinsic to membrane	cellular_component	0.0052322
GO:0006928	cellular component movement	biological_process	0.005331
GO:0034220	ion transmembrane transport	biological_process	0.005509

Supplementary Table 6 List of the top 50 upregulated GO terms following HPIP deficiency

GO accession	Description	Term type	P-value
GO:0003333	amino acid transmembrane transport	biological_process	0.001246
GO:0008839	4-hydroxy-tetrahydrodipicolinate reductase	molecular_function	0.001307
GO:0007050	cell cycle arrest	biological_process	0.001702
GO:0015171	amino acid transmembrane transporter activity	molecular_function	0.001948
GO:0016628	oxidoreductase activity, acting on the CH-CH group of donors, NAD or NADP as acceptor	molecular_function	0.002114
GO:0006865	amino acid transport	biological_process	0.002243
GO:0009085	lysine biosynthetic process	biological_process	0.002417
GO:0009089	lysine biosynthetic process via diaminopimelate	biological_process	0.002417
GO:0046451	diaminopimelate metabolic process	biological_process	0.002417
GO:0030291	protein serine/threonine kinase inhibitor activity	molecular_function	0.0025
GO:0005581	collagen	cellular_component	0.00321
GO:0005669	transcription factor TFIID complex	cellular_component	0.003909
GO:0042168	heme metabolic process	biological_process	0.003914
GO:0005890	sodium: potassium-exchanging ATPase complex	cellular_component	0.006228
GO:0004861	cyclin-dependent protein serine/threonine kinase inhibitor activity	molecular_function	0.006674
GO:0006553	lysine metabolic process	biological_process	0.006836
GO:0019207	kinase regulator activity	molecular_function	0.008223
GO:0019887	protein kinase regulator activity	molecular_function	0.008223
GO:0005201	extracellular matrix structural constituent	molecular_function	0.009847
GO:0019031	viral envelope	cellular_component	0.011276
GO:0036338	viral membrane	cellular_component	0.011276
GO:0004860	protein kinase inhibitor activity	molecular_function	0.013206
GO:0019210	kinase inhibitor activity	molecular_function	0.013206
GO:0005634	nucleus	cellular_component	0.015638
GO:0016591	DNA-directed RNA polymerase II, holoenzyme	cellular_component	0.015698

GO accession	Description	DEG-item	P-value
GO:0034227	tRNA thio-modification	biological_process	0.016147
GO:0004677	DNA-dependent protein kinase activity	molecular_function	0.016323
GO:0000275	mitochondrial proton-transporting ATP synthase complex, catalytic core F(1)	cellular_component	0.016956
GO:0051537	2 iron, 2 sulfur cluster binding	molecular_function	0.017356
GO:0005342	organic acid transmembrane transporter activity	molecular_function	0.017835
GO:0046943	carboxylic acid transmembrane transporter activity	molecular_function	0.017835
GO:0045786	negative regulation of cell cycle	biological_process	0.018177
GO:0008514	organic anion transmembrane transporter activity	molecular_function	0.018306
GO:0003747	translation release factor activity	molecular_function	0.018436
GO:0008079	translation termination factor activity	molecular_function	0.018436
GO:0016538	cyclin-dependent protein serine/threonine kinase regulator activity	molecular_function	0.019825
GO:0042440	pigment metabolic process	biological_process	0.020625
GO:0016840	carbon-nitrogen lyase activity	molecular_function	0.021081
GO:0016627	oxidoreductase activity, acting on the CH-CH group of donors	molecular_function	0.022356
GO:0000428	DNA-directed RNA polymerase complex	cellular_component	0.024299
GO:0030880	RNA polymerase complex	cellular_component	0.024299
GO:0055029	nuclear DNA-directed RNA polymerase complex	cellular_component	0.024299
GO:0009976	tocopherol cyclase activity	molecular_function	0.024379
GO:0043648	dicarboxylic acid metabolic process	biological_process	0.02503
GO:0015849	organic acid transport	biological_process	0.02637
GO:0046942	carboxylic acid transport	biological_process	0.02637
GO:0015711	organic anion transport	biological_process	0.026964
GO:0043231	intracellular membrane-bounded organelle	cellular_component	0.027067
GO:0043227	membrane-bounded organelle	cellular_component	0.027677
GO:0019725	cellular homeostasis	biological_process	0.028132

Supplementary Table 7 List of the top 20 downregulated terms of KEGG pathway analysis following HPIP deficiency

Term	Database	Control number	$HPIP^{-/-}$ number	P-value
Focal adhesion	KEGG PATHWAY	207	26	0.000106
ECM-receptor interaction	KEGG PATHWAY	87	15	0.000155
DNA replication	KEGG PATHWAY	36	8	0.001313
Proteoglycans in cancer	KEGG PATHWAY	204	21	0.004333
Axon guidance	KEGG PATHWAY	127	15	0.004816
Wnt signaling pathway	KEGG PATHWAY	140	16	0.004897
Regulation of actin cytoskeleton	KEGG PATHWAY	215	21	0.007413
Gap junction	KEGG PATHWAY	89	11	0.010924
TGF-beta signaling pathway	KEGG PATHWAY	80	10	0.013897
Melanoma	KEGG PATHWAY	71	9	0.017703
Calcium signaling pathway	KEGG PATHWAY	180	17	0.019604
Malaria	KEGG PATHWAY	49	7	0.020509
Protein processing in endoplasmic reticulum	KEGG PATHWAY	168	16	0.021649
PI3K-Akt signaling pathway	KEGG PATHWAY	347	28	0.021814
Glycosaminoglycan biosynthesis - chondroitin sulfate / dermatan sulfate	KEGG PATHWAY	20	4	0.029236
Pathways in cancer	KEGG PATHWAY	327	26	0.030457
Amoebiasis	KEGG PATHWAY	109	11	0.036923
Complement and coagulation cascades	KEGG PATHWAY	69	8	0.037285
Rap1 signaling pathway	KEGG PATHWAY	211	18	0.037748
cGMP-PKG signaling pathway	KEGG PATHWAY	167	15	0.038544

Supplementary Table 8 List of the top 20 upregulated terms of KEGG pathway analysis following HPIP deficiency

Term	Database	Control number	$HPIP^{-/-}$ number	P-value
p53 signaling pathway	KEGG PATHWAY	68	12	2.81E-05
Small cell lung cancer	KEGG PATHWAY	86	11	0.000739
Focal adhesion	KEGG PATHWAY	207	16	0.007151
Viral carcinogenesis	KEGG PATHWAY	206	15	0.014457
Pathways in cancer	KEGG PATHWAY	327	21	0.01536
Mineral absorption	KEGG PATHWAY	51	6	0.016392
HIF-1 signaling pathway	KEGG PATHWAY	106	9	0.023286
Chronic myeloid leukemia	KEGG PATHWAY	73	7	0.0252
Pentose phosphate pathway	KEGG PATHWAY	28	4	0.027022
FoxO signaling pathway	KEGG PATHWAY	134	10	0.035844
Glutathione metabolism	KEGG PATHWAY	49	5	0.044485
Apoptosis	KEGG PATHWAY	86	7	0.050726
ErbB signaling pathway	KEGG PATHWAY	87	7	0.053185
Protein digestion and absorption	KEGG PATHWAY	89	7	0.058322
Melanoma	KEGG PATHWAY	71	6	0.059114
Sulfur relay system	KEGG PATHWAY	10	2	0.067379
Sulfur metabolism	KEGG PATHWAY	10	2	0.067379
Aldosterone-regulated sodium reabsorption	KEGG PATHWAY	39	4	0.068286
Shigellosis	KEGG PATHWAY	61	5	0.089157
Phosphatidylinositol signaling system	KEGG PATHWAY	81	6	0.093956

Supplementary Table 9 List of the top 50 ChIP-seq assay peaks associated Gene Ontology (GO) terms

GO accession	Description	Term type	P-value
GO:0007275	multicellular organismal development	Biological process	5.79E-32
GO:0048731	system development	Biological process	1.98E-31
GO:0048856	anatomical structure development	Biological process	2.36E-30
GO:0032502	developmental process	Biological process	6.89E-30
GO:0044767	single-organism developmental process	Biological process	4.93E-29
GO:0030154	cell differentiation	Biological process	1.33E-22
GO:0007399	nervous system development	Biological process	4.03E-21
GO:0048869	cellular developmental process	Biological process	2.17E-20
GO:0044707	single-multicellular organism process	Biological process	4.18E-20
GO:0032501	multicellular organismal process	Biological process	1.65E-19
GO:0048513	organ development	Biological process	1.26E-18
GO:0022008	neurogenesis	Biological process	6.42E-18
GO:0051239	regulation of multicellular organismal process	Biological process	1.35E-16
GO:0007267	cell-cell signaling	Biological process	9.49E-16
GO:0048699	generation of neurons	Biological process	1.71E-15
GO:0030182	neuron differentiation	Biological process	4.33E-15
GO:0048468	cell development	Biological process	1.83E-14
GO:0007268	synaptic transmission	Biological process	3.64E-14
GO:0023051	regulation of signaling	Biological process	3.18E-13
GO:2000026	regulation of multicellular organismal development	Biological process	5.19E-13
GO:0009653	anatomical structure morphogenesis	Biological process	9.00E-13
GO:0060284	regulation of cell development	Biological process	4.74E-12
GO:0009888	tissue development	Biological process	6.11E-12
GO:0032879	regulation of localization	Biological process	6.92E-12
GO:0010646	regulation of cell communication	Biological process	6.95E-12

GO accession	Description	DEG-item	P-value
GO:0050793	regulation of developmental process	Biological process	9.23E-12
GO:0048518	positive regulation of biological process	Biological process	9.91E-12
GO:0045595	regulation of cell differentiation	Biological process	1.50E-11
GO:0051960	regulation of nervous system development	Biological process	2.27E-11
GO:0051094	positive regulation of developmental process	Biological process	3.04E-11
GO:0040011	locomotion	Biological process	7.87E-11
GO:0016477	cell migration	Biological process	9.35E-11
GO:0050767	regulation of neurogenesis	Biological process	9.66E-11
GO:0007166	cell surface receptor signaling pathway	Biological process	1.51E-10
GO:0022836	gated channel activity	Molecular function	1.53E-10
GO:0023052	signaling	Biological process	4.42E-10
GO:0044700	single organism signaling	Biological process	5.46E-10
GO:0044459	plasma membrane part	Cellular component	6.17E-10
GO:0044699	single-organism process	Biological process	7.15E-10
GO:0035295	tube development	Biological process	7.62E-10
GO:0005216	ion channel activity	Molecular function	7.72E-10
GO:0009966	regulation of signal transduction	Biological process	7.85E-10
GO:0007610	behavior	Biological process	8.32E-10
GO:0045202	synapse	Cellular component	1.10E-09
GO:0048870	cell motility	Biological process	1.13E-09
GO:0051674	localization of cell	Biological process	1.13E-09
GO:0042127	regulation of cell proliferation	Biological process	1.23E-09
GO:0009887	organ morphogenesis	Biological process	1.35E-09
GO:0051240	positive regulation of multicellular organismal process	Biological process	1.37E-09
GO:0031226	intrinsic component of plasma membrane	Cellular component	1.45E-09

Supplementary Table 10 Sequences of DNA and RNA Oligonucleotides

Name	Forward (5’→3’)	Reverse (5’→3’)
**Primer sequences for real-time quantitative RT-PCR**		
**COL2A1**	CAGAAAGGAGAACCTGGAGATA	AGGGGGACCAGGGGGACCTG
**ACAN**	CAATGAGACCTATGATGTGTACT	CTGCGGTCCGCCAGCCAGCC
**HPIP (m)**	GCCGAGTTGCAGGTCTTTCCA	AGGTCTTGCCCTGCTGCAGG
**β-actin (m)**	CTCTGGTCGTACCACAGGCAT	GTCACGCACGATTTCCCTCTCA
**THBS1**	ACGGAGTTCAGTACAGAAATAAC	CTCGTAGAACAGGAGGTCCACT
**STEAP4**	CAACCTCAAAATCAATCAATATC	GGAGTAAGTCCAAGATTACGAAC
**SPECC1**	ATTAACAGGCTTCGAAGTGAACT	CAAGATAGATCAGTTTCTCCTTC
**CACNB4**	CATTCCAAGTCCACTCAGATTGG	AACACCACCGGACGCATTGACG
**HSPA2**	CGGCGAGAAGAACGTGCTCATC	CGCGCTCGCAAGCGGTGCGC
**LAMA4**	AGTGTAGGAATTGCTTACGCAAC	CAGTCAGGTCCCAGACGCACTT
**TPM3**	GATGAGAGTGAGAGAGGTATGAA	CTCCAGCTCAGAACACTTAGAC
**GOLGB1**	ATGCAACAGAAATTGAGGGTGC	AGAATCTTGGACTCCTCTCTTT
**LAMA3**	CCATGCCAGCAACTGTTACTAT	CCGCATGCTCAGGGTCACAGC
**PIK3R1**	ACGCTATCTCCTGGACTTACCAA	CATTCAACAGATTTTTGCTGGAGG
**B3GNT9**	ACCTGCTTATTGCTGTCAAGT	GCATACGCAAGGCTCTCGGC
**HTRA3**	CTGAGCAGCCCGCGCTACAAG	GTAGCTGCACCTTGAGCTGCTG
**MFAP2**	CCACTATAGCGACCAGATCGAC	GGCCTGTGTATGGAGTAGAGG
**GDF5**	CAGCCGGCCGCCTTGCTGGA	CCAAACACCAGGAACAGGGCT
**IGFBP4**	CAAGGCGTGTGCATGGAGCTG	GAGCCCTGGGGCACAGGCCG
**IGFBP5**	GAAAAGAGCTACCGCGAGCAAGT	AGTGTTCTCGGCTCCCCCGAC
**IGFBP6**	GAAGCTGAGGGCTGTCTCAGGA	ACATCCTGTGGGCGGGCAGTG
**FGF2**	ACTTCAAGGACCCCAAGCGGCT	CCGTAACACATTTAGAAGCCAG
**TNC**	CGACTGCAATGACCAGGGCAAG	CTCCACGCATCGTCCACGGTT
**FBLN1**	CTACAGTGCAAGAGTGGCTTTA	CAGCAGGTGGCGCGCACTCG
**ELN**	GCAGCAGCCGCTAAGGCAGC	GGCTGCCTTTGCAGCTGCCTTA
**ITGA10**	CAATGCCCCATGTGCCAAGGG	CTGAGGCTGGAATGAAGCATCC
**POSTN**	AACCACCTTCACGGATCTTGTG	GAGCTGTTTGCCTCCGATGGTTT
**DCN**	GACTTTATCTGTCCAAGAATCAGCT	CGGATGTAGGAGAGCTTCTTCAT
**VCAM1**	CAGAAATCGAGATGAGTGGTGGC	GCTTTTCCAGTATCTTCAATGGTA
**HPIP (h)**	CTGTGGAGAACCAGGCTGGGG	AGCTGGGCATTCTCCTCCTCT
**IRF-2**	CCAAAACATGGAAGGCGAATTT	AGTGGTCACCTCTACAACTTG
**β-actin (h)**	ATCACCATTGGCAATGAGCG	TTGAAGGTAGTTTCGTGGAT
