# Supplementary material for: Hematopoietic PBX-interacting protein mediates cartilage degeneration during the pathogenesis of osteoarthritis
Source: Nat Commun. 2019 Jan 18;10:313. doi: 10.1038/s41467-018-08277-5 (PMC6338798; doi:10.1038/s41467-018-08277-5)
Supplement: Supplementary file 2 — Reporting Summary [file 41467_2018_8277_MOESM2_ESM.pdf]

nature research

Corresponding author(s): Yan Wang, Qinong Ye, William J. Maloney, Zheng Wang

Last updated by author(s): Dec 9, 2018

Reporting Summary

Nature Research wishes to improve the reproducibility of the work that we publish. This form provides structure for consistency and transparency in reporting. For further information on Nature Research policies, see Authors & Referees and the Editorial Policy Checklist.

Statistics

For all statistical analyses, confirm that the following items are present in the figure legend, table legend, main text, or Methods section.

n/a	Confirmed	
☐	☒	The exact sample size (*n*) for each experimental group/condition, given as a discrete number and unit of measurement
☐	☒	A statement on whether measurements were taken from distinct samples or whether the same sample was measured repeatedly
☐	☒	The statistical test(s) used AND whether they are one- or two-sided<br>*Only common tests should be described solely by name; describe more complex techniques in the Methods section.*
☐	☒	A description of all covariates tested
☐	☒	A description of any assumptions or corrections, such as tests of normality and adjustment for multiple comparisons
☐	☒	A full description of the statistical parameters including central tendency (e.g. means) or other basic estimates (e.g. regression coefficient) AND variation (e.g. standard deviation) or associated estimates of uncertainty (e.g. confidence intervals)
☐	☒	For null hypothesis testing, the test statistic (e.g. *F*, *t*, *r*) with confidence intervals, effect sizes, degrees of freedom and *P* value noted<br>*Give P values as exact values whenever suitable.*
☒	☐	For Bayesian analysis, information on the choice of priors and Markov chain Monte Carlo settings
☐	☒	For hierarchical and complex designs, identification of the appropriate level for tests and full reporting of outcomes
☐	☒	Estimates of effect sizes (e.g. Cohen's *d*, Pearson's *r*), indicating how they were calculated

Our web collection on statistics for biologists contains articles on many of the points above.

Software and code

Policy information about availability of computer code

Data collection	The gene expression data collection can be found in Gene Expression Omnibus (GEO) (http://www.ncbi.nlm.nih.gov/gen/)
Data analysis	Three-dimensional model visualization software (CTVol v2.0) was used for µCT analysis.<br>Indexes of the reference genome were built using Bowtie v2.0.6 and paired-end clean reads were aligned to the reference genome using TopHat v2.0.9.<br>HTSeq V0.6.1 was used to count the read numbers mapped for each gene.<br>A differential expression analysis was performed using the DESeq R package (1.10.1).<br>GO enrichment analysis of differentially expressed genes was implemented by the GOseq R Package.<br>KOBAS software was used to test the statistical enrichment of differential expression genes in KEGG pathways.<br>All analyses were performed using SPSS software 17.0 or GraphPad PRISM 6 (GraphPad).

For manuscripts utilizing custom algorithms or software that are central to the research but not yet described in published literature, software must be made available to editors/reviewers. We strongly encourage code deposition in a community repository (e.g. GitHub). See the Nature Research guidelines for submitting code & software for further information.

Data

Policy information about availability of data

All manuscripts must include a data availability statement. This statement should provide the following information, where applicable:

- Accession codes, unique identifiers, or web links for publicly available datasets
- A list of figures that have associated raw data
- A description of any restrictions on data availability

All relevant data is available from the authors. The RNA-seq data and the complete ChIP-seq datasets in this study was deposited at Gene Expression Omnibus (GEO) ([http://www.ncbi.nlm.nih.gov/gen/]) under accession ID GSE 100312.

Field-specific reporting

Please select the one below that is the best fit for your research. If you are not sure, read the appropriate sections before making your selection.

☒ Life sciences ☐ Behavioural & social sciences ☐ Ecological, evolutionary & environmental sciences

For a reference copy of the document with all sections, see nature.com/documents/nr-reporting-summary-flat.pdf

Life sciences study design

All studies must disclose on these points even when the disclosure is negative.

Sample size	The sample sizes of each set of animals were determined according to previous studies performed by our group and other scholars and were fixed in a prospective manner.
Data exclusions	One mouse in the control group died before the sample collection and thus were excluded for statistical analysis.
Replication	All in vitro experiments were performed in triplicate unless specified in the Figure legends. The detailed replication of each experiments has been provided in Figure Legend. All attempts at replication were successful for all experiments.
Randomization	Animals with the same genotype and similar baseline values were randomly assigned to the sham and surgery groups or the vehicle groups.
Blinding	The experimenters were blinded to the animal genotype and grouping information.

Reporting for specific materials, systems and methods

We require information from authors about some types of materials, experimental systems and methods used in many studies. Here, indicate whether each material, system or method listed is relevant to your study. If you are not sure if a list item applies to your research, read the appropriate section before selecting a response.

Materials & experimental systems

n/a	Involved in the study	
☐	☒	Antibodies
☐	☒	Eukaryotic cell lines
☒	☐	Palaeontology
☐	☒	Animals and other organisms
☐	☒	Human research participants
☒	☐	Clinical data

Methods

n/a	Involved in the study	
☐	☒	ChIP-seq
☒	☐	Flow cytometry
☒	☐	MRI-based neuroimaging

Antibodies

Antibodies used	The detailed information of all antibodies used in our present study has been provided in the METHODS. anti-HPIP (Proteintech, 1:50, 12102-1-AP), anti-LEF1 (Abcam, 1:50, ab137872), H3K4ac (Abcam, 1:50, ab176799), H3K9ac (Abcam, 1:50, ab10812), H3K14ac (Abcam, 1:50, ab52946), H3K56ac (Abcam, 1:25, ab71956),anti-HPIP (1:50, Proteintech, 12102-1-AP), anti-LEF1 (Abcam, 1:200, ab137872), anti-CCND1 (Abcam, 1:2 000, ab134175), anti-c-Myc (Abcam, 1:1 000, ab32072), anti-CUL1 (Abcam, 1:200, ab75817), anti-WNT9A (Abcam, 1:100, ab125957), anti-FZD1 (Abcam, 1:50, ab71342), anti-LRP5 (Abcam, 1:50, ab36121).
Validation	The antibodies were validated by western blot (correct molecular weight, expected response to physiological stimuli, overexpression and/or knockdown of the target).

Eukaryotic cell lines

Policy information about cell lines

Cell line source(s)	The HEK293T cells were purchased from the Type Culture Collection of the Chinese Academy of Sciences (Shanghai, China).
Authentication	The HEK293T cell lines were verified by short tandem-repeat DNA profiling before the study.

Mycoplasma contamination	All cell lines tested negative for mycoplasma contamination.
Commonly misidentified lines (See ICLAC register)	No commonly misidentified cells were used in this study.

Animals and other organisms

Policy information about studies involving animals; ARRIVE guidelines recommended for reporting animal research

Laboratory animals	The detailed information about animals and animal-derived materials used in this study has been provided in our METHODS section. HPIPf/f mice were generated by Cyagen Biosciences Inc. Col2a1-CreERT2 mice (Stock Number: 006774) were obtained from Jackson Laboratories (Bar Harbor, ME, USA). Both HPIPf/f and Col2a1-CreERT2 mice were in a C57BL/6J background. To generate Col2a1-CreERT2; HPIPf/f mice, HPIPf/f mice were mated with Col2a1-CreERT2 mice to obtain Col2a1-CreERT2; HPIPf/+ mice, which were then mated with HPIPf/f mice. Eight-week-old Col2a1-CreERT2; HPIPf/f mice and their HPIPf/f littermates were injected intraperitoneally with tamoxifen (Sigma, St. Louis, MO, USA; 100 µg/g body weight) daily for 5 days before surgical induction of OA. Mice underwent ACLT surgical transection of the right knee to induce mechanical instability and create an experimental OA model. Sham operations were performed on control mice. We analyzed Col2a1-CreERT2; HPIPf/f mice and their HPIPf/f littermates 4 and 8 weeks after surgery. All mice used in the OA evaluation were males to avoid any potential postmenopausal bone loss effect and were maintained under pathogen-free conditions. All experimental procedures were approved by the Institutional Animal Care and Research Advisory Committee of the General Hospital of the People's Liberation Army.
Wild animals	The study did not involve wild animals.
Field-collected samples	The study did not involve samples collected form the field.
Ethics oversight	All experimental procedures were approved by the Institutional Animal Care and Research Advisory Committee of the General Hospital of the People's Liberation Army.

Note that full information on the approval of the study protocol must also be provided in the manuscript.

Human research participants

Policy information about studies involving human research participants

Population characteristics	The clinical and demographic characteristics of the study population are shown in Supplementary Table 1.
Recruitment	OA was macroscopically diagnosed according to the Modified Outerbridge Classification. Articular cartilage samples were collected from 118 patients with knee OA who underwent knee arthroplasty surgery.
Ethics oversight	All human studies were conducted with informed consent of the patients and approval of the Institutional Ethics Review Board of the General Hospital of the People's Liberation Army (Beijing, China).

Note that full information on the approval of the study protocol must also be provided in the manuscript.

ChIP-seq

Data deposition

☒ Confirm that both raw and final processed data have been deposited in a public database such as GEO.

☒ Confirm that you have deposited or provided access to graph files (e.g. BED files) for the called peaks.

Data access links *May remain private before publication.*	https://www.ncbi.nlm.nih.gov/geo/query/acc.cgi?acc=GSE100312
Files in database submission	GSE100311 HPIP controls osteoarthritis cartilage degeneration [ChIP-seq]
Genome browser session (e.g. UCSC)	No longer applicable

Methodology

Replicates	2 biological replicates of the Chip-seq in cells. Individual peak calls for each replicate with SPP, IDR (irreproducable discovery rate) processed peaks in a separate file.
Sequencing depth	Libraries were PCR-amplified for 16 cycles, fragments with size of 300 bp were processed using the Covaris Focused-ultrasonicators (Covaris, Inc. S220). Sequencing was performed using the Hi-Seq 2500 (Illumina) platform at Beijing Novel Bioinformatics Co., Ltd. (https://en.novogene.com/). ChIP-seq data was aligned to the hg19 genomes using bowtie-0.12.9. The basic quality statistics analysis of raw reads was evaluated by the FastQC software. Raw reads were then trimmed to remove the duplicate reads and reads that were matched with the adaptor sequence and the base whose mass value of the 3'tail base number that was less than 20. If the remaining sequence length was not shorter than 18nt, it will be retained. Only tags mapped uniquely to the genome were considered for further analysis.

Antibodies	Anti-HPIP were used from Proteintech (12102-1-AP)
Peak calling parameters	Genomic binding peaks for HPIP were identified using the findPeaks command from HOMER (http://homer.salk.edu/homer/) with 8-fold enrichment over the input sample. Peaks were annotated using the annotatePeaks command.
Data quality	We select 13 as the threshold for mapping quality (MAPQ) to ensure the high quality.
Software	Indexes of the reference genome were built using Bowtie v2.0.6 and paired-end clean reads were aligned to the reference genome using TopHat v2.0.9.<br>HTSeq V0.6.1 was used to count the read numbers mapped for each gene.<br>A differential expression analysis was performed using the DESeq R package (1.10.1).<br>GO enrichment analysis of differentially expressed genes was implemented by the GOseq R Package.<br>KOBAS software was used to test the statistical enrichment of differential expression genes in KEGG pathways.
